# High-Resolution Magic Angle Spinning (HR-MAS) NMR-Based Fingerprints Determination in the Medicinal Plant *Berberis laurina*

**DOI:** 10.3390/molecules25163647

**Published:** 2020-08-11

**Authors:** Sher Ali, Gul Badshah, Caroline Da Ros Montes D’Oca, Francinete Ramos Campos, Noemi Nagata, Ajmir Khan, Maria de Fátima Costa Santos, Andersson Barison

**Affiliations:** 1 NMR Lab, Department of Chemistry, Federal University of Paraná, Curitiba 81530-900, PR, Brazil; gulbadshahhum1188@gmail.com (G.B.); caroline.ros@hotmail.com (C.D.R.M.D.); nnagata@ufpr.br (N.N.); fatima.mcs2529@gmail.com (M.d.F.C.S.); 2 Department of Pharmacy, Federal University of Paraná, Curitiba 80210-170, PR, Brazil; francampos@ufpr.br; 3 School of Packaging, Michigan State University, East Lansing, MI 48824-1223, USA; ajmirkhan33@yahoo.com; 4 Institute of Chemistry, University of São Paulo, São Paulo 05508-000, SP, Brazil

**Keywords:** *Berberis laurina*, metabolomic analysis, HR-MAS NMR, metabolites, chemometric analysis

## Abstract

*Berberis laurina* (Berberidaceae) is a well-known medicinal plant used in traditional medicine since ancient times; however, it is scarcely studied to a large-scale fingerprint. This work presents a broad-range fingerprints determination through high-resolution magical angle spinning (HR-MAS) nuclear magnetic resonance (NMR) spectroscopy, a well-established flexible analytical method and one of most powerful “omics” platforms. It had been intended to describe a large range of chemical compositions in all plant parts. Beyond that, HR-MAS NMR allowed the direct investigation of botanical material (leaves, stems, and roots) in their natural, unaltered states, preventing molecular changes. The study revealed 17 metabolites, including caffeic acid, and berberine, a remarkable alkaloid from the genus *Berberis* L. The metabolic pattern changes of the leaves in the course of time were found to be seasonally dependent, probably due to the variability of seasonal and environmental trends. This metabolites overview is of great importance in understanding plant (bio)chemistry and mediating plant survival and is influenceable by interacting environmental means. Moreover, the study will be helpful in medicinal purposes, health sciences, crop evaluations, and genetic and biotechnological research.

## 1. Introduction

**Figure 1:**
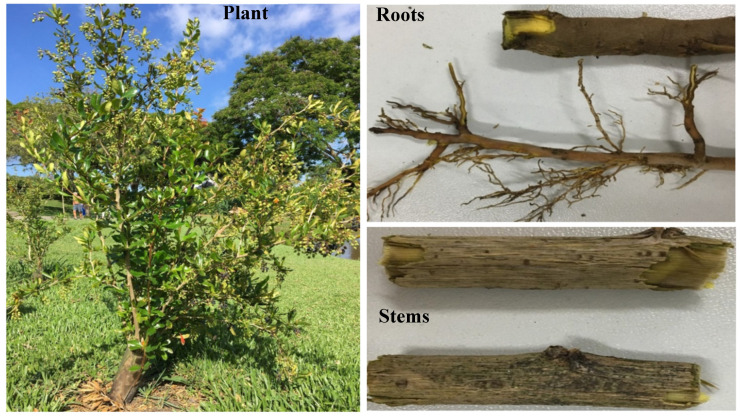
A representative specimen of *Berberis laurina* Billb. and its parts. The photos are available at http://www.ufrgs.br/fitoecologia/florars/open_sp.php?img=11160.

The family Berberidaceae consists of about 13 genera and 600 species, including the commonly known genus *Berberis* L., which is considered the main contributor with 500 species, including the species *Berberis laurina* Billb. [1]. The usual physiognomies nature of such genus are their highly spiny, deciduous shrubs or small woody trees with characteristic yellow flowers. This genus is well-known as a pharmacological source in traditional medicine systems since ancient times [1]. The species *B. laurina* Billb. (Figure 1) is frequently distributed in the Northern hemisphere, some Asian countries [1], and in some South American countries, particularly in the south and southeast of Brazil, as well as in Argentina, Uruguay, and Paraguay, where it is known as *Espinho-de-São-João Berbéris-da-terra*, *Quina-cruzeiro*, *Uva-de-espinho*, *Espina-amarilla*, and *Palo-amarillo* [2]. Although there are a few missing statements of the complete chemical profiles related to *B. laurina* Billb., since, this gap has been correspondingly completed in the current work focused on the aerial (leaves and stems) and underground (roots) parts of the species.

Nuclear magnetic resonance (NMR) is worldwide well-stablished spectroscopic technique that allows to obtain information related to the genotype, phenotype, and intra- and interorganism classifications based on its origin and biological importance, environmental toxicity, and pollution [3,4,5,6,7,8,9,10]. In such, NMR spectroscopy is widely used in multidisciplinary “omics”, such as metabolomics, metabolic profiling, fingerprinting, and phenotyping [11,12,13,14], as well as in identification and structural determination of organic compounds in various samples such as food [4], ice [8], serum [10], environmental [15], material science [16], and water [17]. In addition, high-resolution magic angle spinning (HR-MAS) is a multipurpose NMR tool allowing the acquisition of NMR data directly from semi-solid (i.e., gel-like) materials (e.g., plant tissues) in their natural, unaltered states, without laborious sample preparation steps, and then preventing changes in the chemical composition during these process [18,19]. Moreover, the HR-MAS NMR technique uses specialized HR-MAS probes that allow to collect high-resolution spectra from heterogeneous samples with remarkably similar spectral resolutions as those observed for homogeneous samples in a liquid state (i.e., solution state).

Due to restricted and low molecular tumbling conditions, botanical samples contain several anisotropic trends such as dipolar (through bonds and space) interactions, magnetic susceptibility, and chemical shift anisotropy [18,20]. These trends directly affect T2 relaxation, which produces a nonuniform shift (line-broadening), also causing low signal-to-noise and resolution in NMR spectra [18,21,22,23]. Dipolar coupling is proportional to the “3cos^2^ θ−1” term in the second-order Legendre polynomial equation: “P_2_(cos^2^ θ) = 1\2(3cos^2^ θ−1)”. Therefore, the line-broadening effects coming from dipolar interactions can be minimized by spinning the sample at high spinning rates at the so-called magic angle (*θ _MAS_* = 54.74°) [18,22]. Additionally, in order to improve spectral resolution [18], the HR-MAS technique is to be applied to the swollen sample in a suitable NMR solvent that provides some molecular motions [19,22]. In addition to the liquid state, HR-MAS NMR has been used in metabolic analyses in human, plant, and food stuff quality managements, genotype, phenotype, and organism cataloging, with interindividual comparisons, environmental toxicity, and pollution [24,25,26,27,28,29,30]. Moreover, crowded spectral overlaps and chemical structure elucidations can be facilitated by mapping all homo- and heteronuclear correlations through multidimensional (nD) NMR approaches [31].

The plants (bio)chemically produce a composite assembly of different features of multiclass, small, organic metabolites as basic needs of energy, protections, and growth [29,32,33]. These molecular assemblies, even though normal growths, are highly affected through irregular environmental conditions. To trace such relationships (plant environments), since periodic investigations of metabolites are useful tools and, also, helpful in understanding the (bio)chemistry and other biological events [34]. Plants can adapt to any (un)suitable environments by rearranging their genetics to molecular outlines and productions to respond to unfeasible environmental impacts [35]. Additionally, this could provide excellent glimpses into chemical and biological research about vital relationships and to the discovery of new chemical entities with potential applications in medicinal chemistry [29]. Excepting metabolic mechanisms, interactive environmental effects to the molecular patterns within plant topology has been limited. In this regard, within plants, the topological order was measured through HR-MAS NMR-based fingerprinting, which was followed by multivariate statistical analysis such as principal component analysis (PCA) approach [11,12]. Since the study was sustained along seven months (October 2018 to April 2019) to spectroscopically and statistically correlate chemical alterations within aerial parts of the individuals in the associated period.

## 2. Results and Discussion

**Figure 2:**
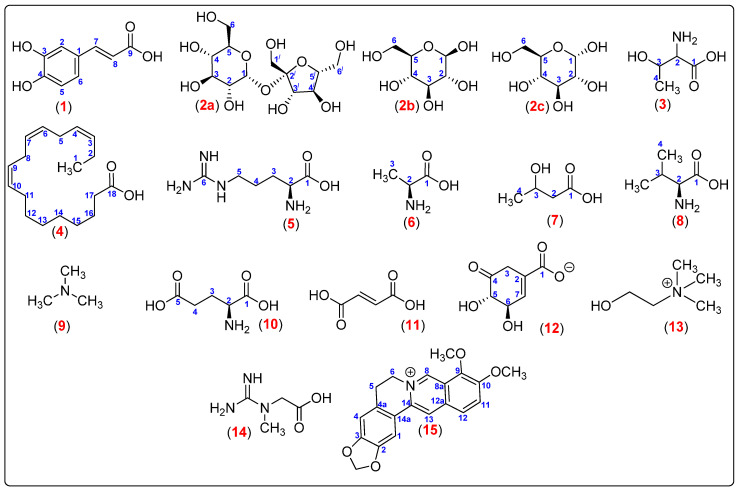
Metabolites identified in the leaves, stems, and roots of *Berberis laurina* (Berberidaceae).

In present work, leaves, roots, and stems of *Berberis laurina* Billb. (Berberidaceae) were directly investigated in their natural state through ^1^H High-Resolution Magic Angle Spinning Nuclear Magnetic Resonance (HR-MAS NMR) approach, without sample pretreatment steps, and then preventing changes in the chemical compositions during extraction and isolation procedures. Following that, as the ^1^H NMR spectra taken in solution were remarkably like to those in semi-solid taken by means of HR-MAS (Figure S1), the liquid-state 2D NMR experiments were performed to facilitate metabolite identifications. In turn, a range of 17 primary and secondary specialized metabolites in all plant parts (leaves, stems, and roots) were detected (Figure 2).

The principal chemical constituents found in the leaves in comparison to stems and roots were caffeic acid (**1**), sucrose (**2a**), β-glucose (**2b**), α-glucose (**2c**), threonine (**3**), fatty acids (Linolenic acid and **4**), arginine (**5**), alanine (**6**), 3-hydroxybutyric acid (**7**), valine (**8**), trimethylamine (**9**), glutamic acid (**10**), fumaric acid (**11**), dihydroxy shikimate (**12**), choline (**13**), creatine (**14**), and berberine (**15**), as shown in Figure 2. In general, fairly to leaves, the stems but, remarkably, the roots were observed rich sources of berberine (**15**) (Figure S2).

### 2.1. ^1^H HR-MAS NMR-Based Chemical Composition of the Leaves of Berberis laurina

**Figure 3:**
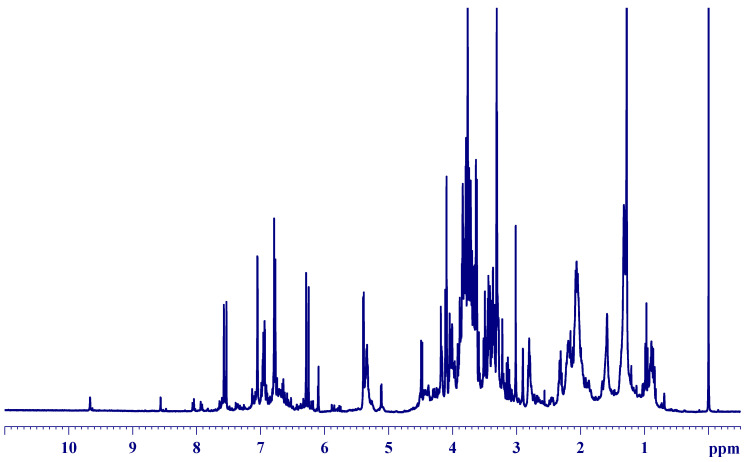
^1^H HR-MAS NMR (ẟ −0.50–11.00) spectrum from the leaves of *Berberis laurina* (400 MHz, ~10 mg swollen in 40-µL CD_3_OD).

**Figure 4:**
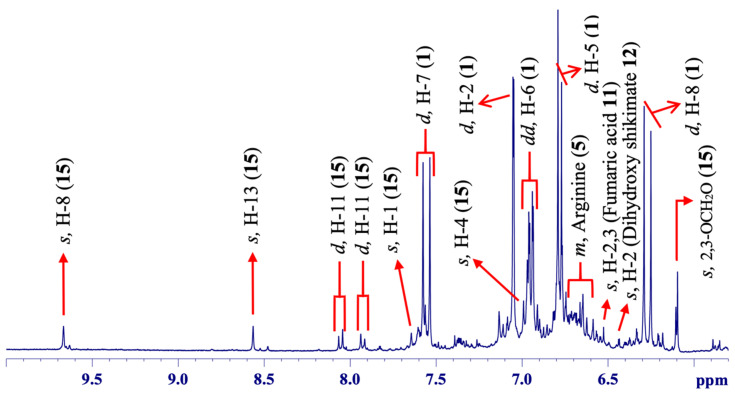
^1^H HR-MAS NMR (ẟ 5.80–10.00) spectrum showing signal annotations from leaves of *Berberis laurina* (400 MHz, ~10 mg swollen in 40-µL CD_3_OD).

**Figure 5:**
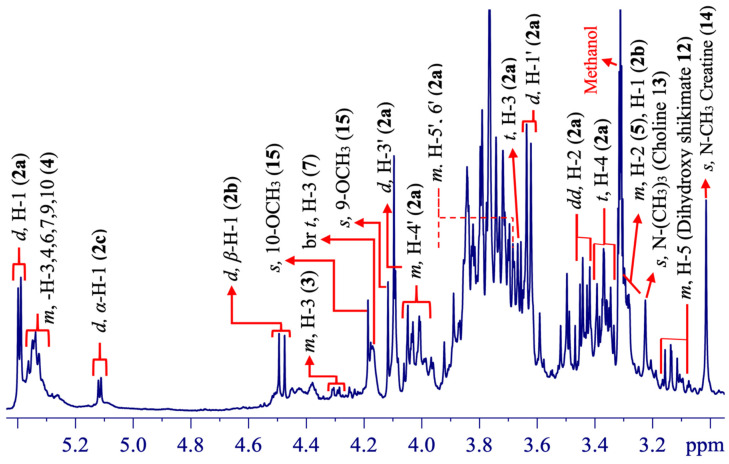
^1^H HR-MAS NMR (ẟ 2.90–5.45) spectrum showing signal annotations from the leaves of *Berberis laurina* (400 MHz, ~10 mg swollen in 40-µL CD_3_OD).

**Figure 6:**
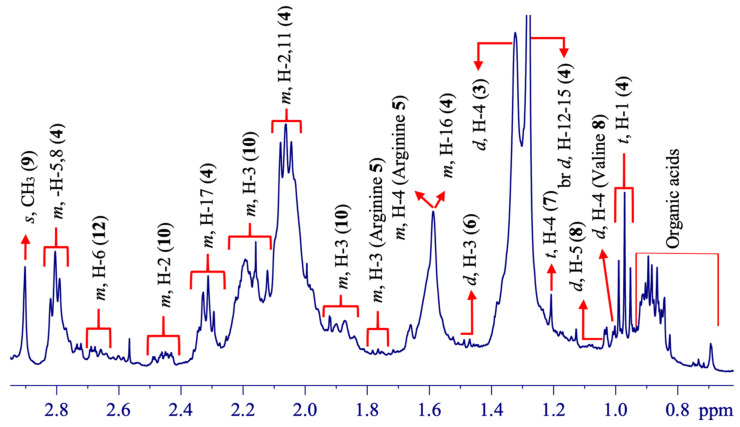
^1^H HR-MAS NMR (ẟ 0.67–2.96) spectrum showing signal annotations from the leaves of *Berberis laurina* (400 MHz, ~10 mg swollen in 40-µL CD_3_OD).

The spectral profile acquired directly from the leaves of *B. laurina* (Figure 3) seemed to be very overlapped and difficult to clearly identify the signals from the chemical compounds. Thus, it was divided into three major segments: the high (aromatic; Figure 4, middle (carbohydrate; Figure 5), and low-frequency region (aliphatic region; Figure 6), which are discussed individually as follows.

In the aromatic region of the spectrum (Figure 4), three chemical components, caffeic acid (**1**), fumaric acid (**11**), and berberine (**15**), with additional signals from other compounds (i.e., arginine **5** and dihydroxy shikimate **12**), were observed.

Caffeic acid (**1**) was detected due to its typical two doublet signals with larger and equal magnitudes of scalar (*J*) couplings representing a *trans*-configuration in the system; one was at ẟ 7.55 (d, ^3^*J*_H-H_ = 15.9 Hz, H-7), and the second was at ẟ 6.27 (d, ^3^*J*_H-H_ = 15.9 Hz, H-8), assigned to the hydrogens on positions 7 and 8 [36]. An intense doublet signal of a small *J*-coupling at ẟ 7.04 (d, ^4^*J*_H-H_ = 1.9 Hz, H-2) was assumed to be an aromatic H-2 *meta*-coupled to H-6 revealed by a double doublet at ẟ 6.95 (dd, ^3,4^*J*_H-H_ = 8.1; 1.9 Hz, H-6). Its splitting pattern showed that it was still *ortho*-coupled to H-5 exposed via a doublet at ẟ 6.76 (d, ^3^*J*_H-H_ = 8.1 Hz, H-5) in the molecular system of **1**. The entire signal assignments for **1** were confirmed based on 2D NMR experiments performed in solution state (Figures S3–S11), as well as previous reported data [36]. Caffeic acid (**1**) is a characteristic metabolic component of the phenylpropanoid or lignin biosynthetic pathway in plants [37,38]. It is chemically a functional metabolite in the plant itself as an antipredator agent, nascent leaves protector, and growth developer, as well as antioxidant, anti-inflammatory, and antiviral and functional in cardiovascular and diabetes diseases [37]. It has been previously described in *Berberis aristata* DC., a plant from the same family of *B. laurina* Billb. [39].

Fumaric acid (**11**) [40] was detected through a typical singlet signal at ẟ 6.54 (s, H-2, and 3), representing both hydrogens at positions 2 and 3 in the molecule. It is a small organic compound involved in the tricarboxylic acid cycle as a basic component for energy storage, and consumed in the biosynthesis of other molecules in plants [41]. This small organic acid is generally used as an additive and antioxidant agent in food products and useful as anti-inflammatory and antibacterial [42,43].

Berberine (**15**), a main alkaloid compound, was identified based on several singlet signals in the high-frequency range of δ 9.67 (s, H-8) and δ 8.56 (s, H-13) [44]. Moreover, two individual doublets of equal *J*-coupling constants were observed in the δ 8.05 (d, ^3^*J*_H-H_ = 9.1 Hz, H-11), representing H-11, *ortho*-coupled to H-12, that appeared at δ 7.93 (d, ^3^*J*_H-H_ = 9.1 Hz, H-12) on the aromatic site in **15**. Additionally, one of the two individual singlets was revealed at δ 7.64 (s, H-1) and the other singlet signal at δ 6.99 (s, H-4). Likewise, a singlet was revealed at δ 6.09 (s), which was assigned to two hydrogen nuclei in a methylene group directly connected to two oxygen atoms (-O-CH_2_-O-). The remaining signals were observed in another segment of the spectrum (Figure 5) at δ 4.11 (s) and 4.18 (s) which were assigned to methoxy hydrogens (-OCH_3_) on positions 9 and 10, respectively. Similarly, the remaining two signals (triplets) were in a highly crowed region of the spectra and were not observed. The entire signal assignments (with minor distinctions in chemical shifts) for **15** were comparative to those in the spectrum recorded from the roots (also stems) of *B. laurina* that provided all signals at higher intensities (discussed below) and were in accordance with those in previously published data [44]. The berberine compound has been identified in the literature, although using several pretreatment sequences such as extraction, isolation, and purification and, so, characterized through some spectroscopic and spectrometric approaches [45]. Additional reports highlighted the presence of alkaloids, terpenoids, flavonoids, sterols, anthocyanins, lignans, vitamins, proteins, lipids, and carotenoids in multiple *Berberis* genera from Berberidaceae [1].

The second segment of the spectrum (Figure 5) showed several superimposed signals related to various chemical components such as carbohydrates (sucrose (**2a**), β-glucose (**2b**), α-glucose (**2c**)), threonine (**3**), fatty acids (**4**), arginine (**5**), 3-hydroxybutyric acid (**7**), dihydroxy shikimate (**12**), choline (**13**), creatine (**14**), and some signals from berberine (**15**).

The presence of sucrose (**2a**), β-glucose (**2b**), and α-glucose (**2c**) were confirmed based on their typical anomeric hydrogen signals, such as doublets at ẟ 5.38 (d, ^3^*J*_H-H_ = 3.8 Hz, α-H in glucose unit), ẟ 4.47 (d, ^3^*J*_H-H_ = 7.8 Hz, β-H), and ẟ 5.12 (d, ^3^*J*_H-H_ *=* 3.7 Hz, α-H). The assignments of carbohydrate contents (**2a**–**c**) were, in comparison to the published data, acquired in methanolic extracts of *Citrus*-type crude drugs of Kijitsu, Touhi, Chimpi, Kippi, and Seihi botanical materials [46]. Carbohydrate contents were essentially distributed and considered the main sources of energy in plants, as evidenced in roots of *Berberis chitria* Buch.-Ham. ex Lindl. of same family Berberidaceae [45].

Threonine (**3**) was detected by a broad and less intense multiplet signal in the range of ẟ 4.29 (*brm*, H-3) [47]. Threonine is a primary metabolite, principal growth regulator and defender in drastic conditions, as well as a nutritional needs promoter in plants [48,49]. A further primary metabolite, the fatty acid (**4**), was detected by certain signals of vinylic hydrogen nuclei appeared at ẟ 5.34 (m, H-3, 4, 6, 7, 9, and 10) [50,51]. Choline (**13**) was observed by a singlet signal for N-(CH_3_)_3_ at ẟ 3.20 (s) [40] and 3-hydroxybutyric acid (**7**) [47] at ẟ 4.18 (*brs*, H-3), plus an additional broad signal at ẟ 1.21 (*brs*, H-4) in Figure 5. Alanine (**6**) was detected by a doublet at ẟ 1.48 (d, ^3^*J*_H-H_ = 7.20 Hz, H-3) [52], dihydroxy shikimate (**12**) was observed (Figure 4, Figure 5 and Figure 6) by a singlet at ẟ 6.38 (s, H-2), and two multiplets at ẟ 3.10 (m, H-5) and ẟ 2.66 (m, H-6) [40].

The singlet signal at ẟ 3.02 (s, 3H) (Figure 5) was assigned to the N-CH_3_ methyl group of creatine (**14**). Although this assignment should be carefully evaluated, since creatine is not considered a compound from the kingdom plantae. There are only few reports, such as in *Phaseolus mungo* (L.) Hepper, *Lens culinaris* Medik., and *Eugenia uniflora* L. [53,54]. Even though, it is remarkably interesting to notice that this same NMR singlet signal was previously described only in *Eugenia uniflora* L. [19,54]. In several works regarding plant tissue investigations through HR-MAS NMR approach, any singlet signal has been described around ẟ 3.02 ppm, excepting for *Eugenia uniflora* L. and, now, *Berberis laurina* Billb.

In addition the third spectral part (Figure 6) showed several signals from fatty acids (**4**), arginine (**5**), alanine (**6**), 3-hydroxybutyric acid (**7**), valine (**8**), trimethylamine (**9**), and glutamic acid (**10**), correspondingly.

The fatty acids (**4**) were confirmed specifically based on a triplet at ẟ 0.97 (^3^*J*_H-H_ = 7.6 Hz) of a linolenic acid counterpart [50,55]. The presence of this fatty acid chain would be suggested as one of the fatty acid acyl chains that constitute the lipophilic membrane of leaves and plays an integral defensive function in oxireduction processes during biochemical cycles of vegetal. Arginine (**5**) was characterized by multiplets observed in both spectral regions (Figure 4, Figure 5 and Figure 6) by ẟ 3.27 (m, H-2), 1.77 (m, H-3), 1.60 (m, H-4), and 1.92 (m, H-5). Similarly, valine (**8**) was identified by a doublet signal at ẟ 1.03 (d, ^3^*J*_H-H_ = 2.7 Hz, H-5), trimethylamine (**9**) by a singlet at ẟ 2.90 (s, N-(CH_3_)_3_), and glutamic acid (**10**) by multiplets at ẟ 2.45 (m, H-2) and 2.0 (m, H-3). In addition, certain signals were observed for already mentioned metabolites declared relatively in the previous spectral ranges (Figure 5). Chemical compounds such as arginine (**5**), alanine (**6**), and 3-hydroxybutyric acid (**7**) were previously observed in mangos during fruit developmental processes [47]. The other primary metabolites such as threonine (**3**), valine (**8**), trimethylamine (**9**), glutamic acid (**10**), dihydroxy shikimate (**12**), and choline (**13**) were previously reported in *Commiphora wightii* [40].

The overall NMR chemical shifts, coupling constants, and related literature to the chemical compounds (**1** to **15**) are shown in Table 1, as well as associated 2D NMR correlation maps are correspondingly given (Figures S3–S11).

### 2.2. ^1^H HR-MAS NMR-Based Chemical Composition of Stems and Roots of Berberis laurina

**Figure 7:**
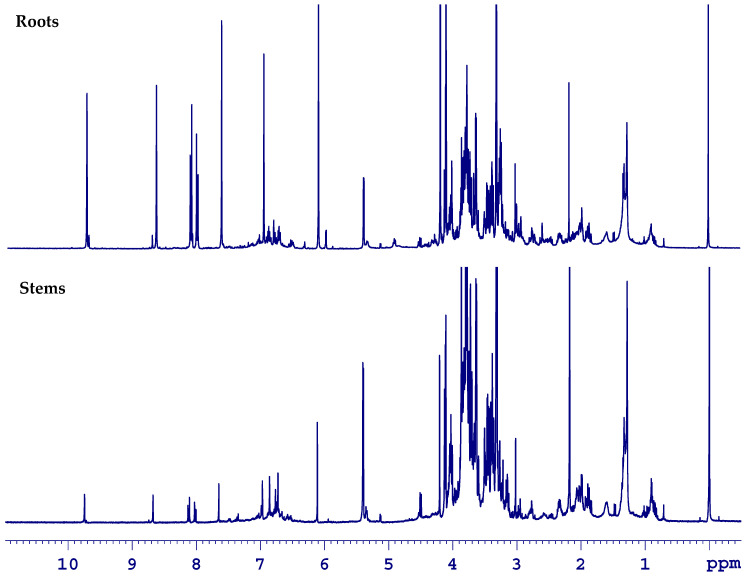
Comparative overview of ^1^H HR-MAS NMR spectra (ẟ −0.50–11.00) from the roots and stems parts of *Berberis laurina* (400 MHz, ~10 mg swollen in 40 µL CD_3_OD).

In same way, the stems and roots of *Berberis laurina* Billb. were analyzed through HR-MAS NMR spectroscopy in their natural, unaltered states. In comparison to leaves, the HR-MAS NMR spectra of the stems and roots presented less overlapped NMR spectra, which are associated with high-intense signals, mainly in those from the roots (Figure 7).

**Figure 8:**
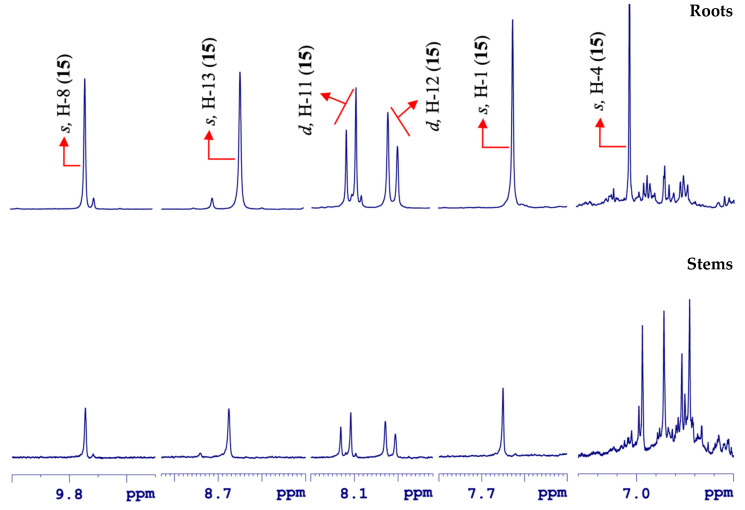
Comparative overview of the amplified aromatic region in the ^1^H HR-MAS NMR spectra from the roots and stems parts in *Berberis laurina* (400 MHz, ~10 mg swollen in 40 µL CD_3_OD).

**Figure 9:**
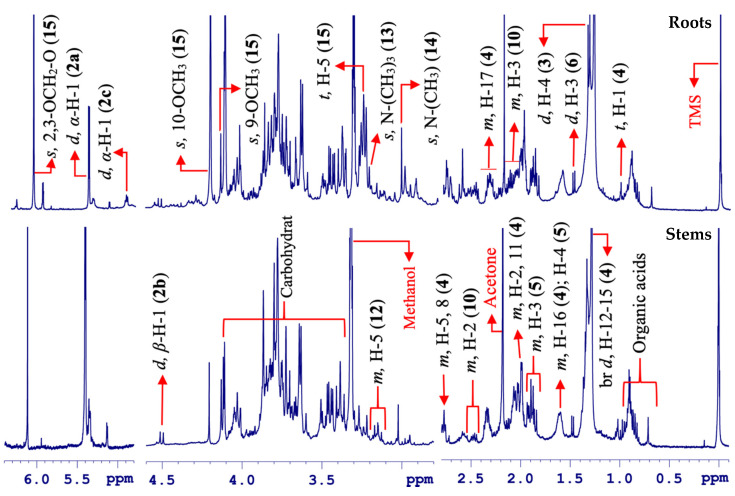
Comparative overview of the amplified olefinic, carbohydrates, and aliphatic regions in the ^1^H HR-MAS NMR spectra from the roots and stems of *Berberis laurina* (400 MHz, ~10 mg swollen in 40-µL CD_3_OD).

In this case, to facilitate the chemical compositional overview for the roots and stems, the spectra were divided into two sections: the high frequency comprising aromatic and olefinic signals and low frequency containing aliphatic ones (Figure 8 and Figure 9).

Berberine (**15**) was initially observed through two singlet signals at δ 9.74 (s, H-8) and 8.65 (s, H-13) [44]. Furthermore, two doublets of equal *J*-coupling constants were observed at δ 8.11 (d, ^3^*J*_H-H_ = 9.1 Hz, H-11) and 8.00 (d, ^3^*J*_H-H_ = 9.1 Hz, H-12) from two mutually *ortho*-coupled hydrogen nuclei on the aromatic ring in the molecule. Additionally, other intense singlets appeared at δ 7.63 (s, H-1) and 6.96 (s, H-4) (Figure 8).

Similarly, the remaining singlets at δ 6.11 (s), 4.20 (s, 3H), and 4.11 (s, 3H) from hydrogen nuclei in the 2-O-CH_2_-O-3, H_3_CO-10, and H_3_CO-9 groups were observed in the aliphatic region. Likewise, two individual triplets of equal *J*-coupling magnitudes were found at δ 4.92 (t, ^3^*J*_H-H_ = 6.3 Hz, H-5) and 3.26 (t, ^3^*J*_H-H_ = 6.3 Hz, H-6), which were mutually coupled due to their coupling patterns and were assigned to H-5 and H-6 (Figure 9).

According to the previous studies, berberine is an alkaloid of strongly yellow color and active as an antifungal, antibacterial, antiviral, cardiovascular, anti-inflammatory, antidiabetes, and other biological activities [56]. The findings from this work allowed to realize that the roots followed by stems of *Berberis laurina* are a rich natural source for the alkaloid berberine (**15**). On the other hand, the aerial parts are an interesting source for caffeic acid.

Other than berberine (**15**), the carbohydrate contents included sucrose (**2a**), which was perceived by means of distinct doublets at δ 5.40 (d, ^3^*J*_H-H_ = 3.8 Hz, H-1 in the glucose unit), the β-glucose (**2b**) by the signal at δ 4.51 (d, ^3^*J*_H-H_ = 7.8 Hz, β-H), and also, α-glucose (**2c**) by a representative doublet at δ 5.14 (d, ^3^*J*_H-H_ = 3.7 Hz, α-H). Less intense signals were observed for vinylic hydrogen nuclei at δ 5.34 (m) and δ 1.28 of methylene (CH_2_) of fatty acids (**4**), with an additional singlet signal at δ 3.02 (s) for creatine (**14**) in the fresh roots and stems of *B. laurina*.

Additionally, the confirmation of all identified chemical compounds in all three parts (leaves, stems, and roots) were based on HR-MAS followed by 2D NMR experiments in a liquid state (Figures S3–S11) and the literature data, as well as online databases such as MetaboLights available at https://www.ebi.ac.uk/metabolights/index and the Biological Magnetic Resonance Data Bank, BMRB, which can be accessed at http://www.bmrb.wisc.edu/. An overview of the complete details related to the identified metabolites in *Berberis laurina* are presented in Table 1.

### 2.3. ^1^H HR-MAS NMR-Based Insight into the Leaves Metabolic Patterns

Plants are natural resources to produce manifold small organic compounds, covering intermediates to final products of multiple intracellular biosynthetic events, which are closely associated to the environmental conditions [32,57]. These small chemical entities are primary and secondary metabolites of distinctive classes of carbohydrates, organic and fatty acids, terpenoids, alkaloids, and phenolic compounds that have several functional properties [29,32,33]. Metabolites are crossing points between plant and environmental trends that are main stimuli to the usual life stages and affective towards the metabolic patterns, as seen in different parts (roots, stems, leaves, and flowers) of several plants [32,58].

To study the metabolic pattern (or fingerprints) associated to the environment and periodical flux, HR-MAS NMR-based fingerprinting analyses were applied to follow the chemical compositions over the time, as well as according to plant topology. In such, the HR-MAS NMR approach was applied to the top, middle, and bottom of *Berberis laurina* during a period of seven months (October 2018 to April of 2019). The quantitative levels of chemical compounds could be traced directly from its HR-MAS NMR spectra. Indeed, the signal intensities were proportional to the amount of chemical compounds in the materials. The plant was cultivated in an open environment, meaning that it was totally exposed to environmental (a)biotic communications.

**Figure 10:**
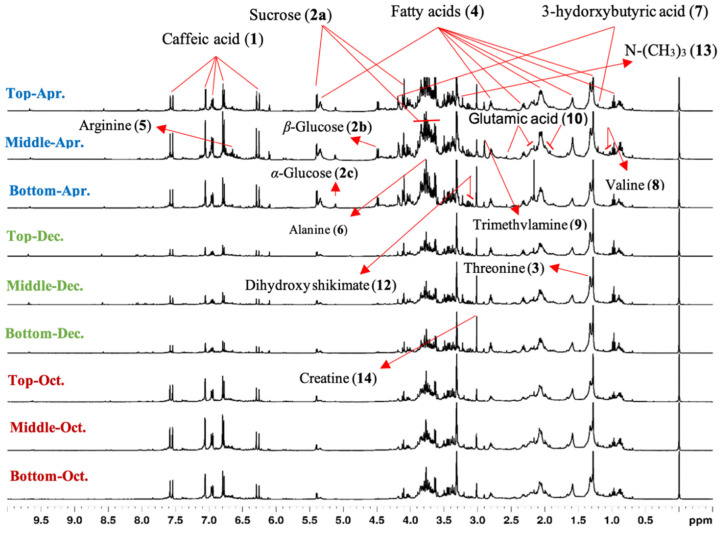
Stacked outline and visual assessment of the ^1^H HR-MAS NMR spectra recorded from the leaves of *Berberis laurina* associated to different times (October 2018 to April 2019).

The visual inspection of HR-MAS NMR spectra disclosed most of the intense signals in the middle leaves, followed by the top and bottom. Substantial increases in the signals’ relative intensities were observed for caffeic acid (**1**), sucrose (**2a**), β-glucose (**2b**), α-glucose (**2c**), and creatine (**14**) metabolites (Figure 10). On the other hand, screening the same spectra in accordance to the fluctuated period (months), manifold signal intensifications could be observed frequently in all metabolites during October 2018 (Spring) and April 2019 (Fall). The quantitative (de)escalation in the plant metabolic profiles was supposed to be due to the interlinked environmental effects on the chemical substances, which have been previously described for other plants [32,59,60].

The comparative spectral profiles (October 2018 to April 2019) demonstrated that those signals for caffeic acid (**1**) were of high intensities in October 2018, while they were downregulated in December 2018 and again upregulated in April 2019 (Figure 10). According to the climate data, November and December 2018 experienced a meaningful reduction in rainfall precipitation, as well as temperatures that started to increase, with the highest one in December 2018 (Figure S12). This means that caffeic acid production may be correlated to water scarcity, or its need decreases in low precipitation seasons. In a holistic overview regarding all seven months (October 2018 to April 2019), it was observed that bottom leaves presented only a small higher average production of caffeic acid (**1**), although they presented the higher dispersion over the time, as well (Figure S13).

The signals for sugar components sucrose (**2a**), β-glucose (**2b**), and α-glucose (**2c**) were of high intensities in April 2019. In March and April 2019, the temperatures started to decrease as well as a reduction in rainfall precipitation was experienced (Figure S12). Although, this correlation must be noticed carefully, since both of them presented relatively high content dispersions over the time, no matter the plant topology (Figure S13).

These up- (and or down) regulations in signal intensities indicate a significant dependence on the environmental conditions, such as rainfall, solar indices, and seasonal and temperature changes [32,59,60].

### 2.4. Principal Component Analysis-Based Metabolic Pattern Discrimination in the Leaves

The comprehensive details regarding the molecular pattern fluctuations revealed by HR-MAS NMR analysis may be furthermore streamlined through multivariate statistical analysis by means of principal component analysis (PCA) [12,61]. In this, features can be retained when multidimensional HR-MAS NMR raw data is mathematically transformed into readable small-dimensional variables, the principal components (PCs). In turns, the main objective performing PCA was to follow the changes in the chemical compositions of aerial parts *Berberis laurina* over the time (month-wise) in a smart way.

As described previously (see Section 2.3), the plant topology was associated to the leaves (top, middle, and bottom). The NMR experiments were conducted in triplicate (*n* = 3 × 9) under the same and uniform experimental conditions. PCA was performed with the aid of a Bruker AMIX software package to a selected spectral range (ẟ 0.60–10.00), excluding unwanted regions such as the residual water signal (ẟ 4.73–5.00), as well as acetone (ẟ 2.14–2.16) and partially deuterated methanol (ẟ 3.29–3.32) residual signals. First of all, the ^1^H MHR-MAS NMR spectra were converted in buckets (binning) by dividing the spectral width into equal small segments 0.04-ppm wide, resulting in a X-sized bucket table equivalent to 28 rows containing NMR spectra (i.e., samples) vs. 174 columns comprising the variables (i.e., NMR chemical shifts). In the generated buckets table, each relative intensity along the rows (i.e., spectra) was normalized based on the total spectral area, while, column-wise (i.e., variables), were submitted to pareto scaling. In contrast to autoscaling and no scale, the pareto scaling method is supposed to be beneficial, particularly in NMR-based fingerprinting approaches, which balance all nonuniform variables by avoiding expected noise and additional artifacts in the spectra [62,63]. In other words, autoscaling means that all columns (i.e., variables) are equally weighted during PCA, although it can overestimate those buckets containing noise. On the other hand, no scaling preserves the natural differences in intensities, although it highlights dominant effects such as high-intensity signals in detriment to those that have a low intensity. Pareto scaling is in between no scaling and autoscaling, without overestimating noisy variations by reducing the relative importance of intense buckets and keeping the data structure partially intact. In the mathematical sense, the pareto scale divides the mean centered variables by the square root of the standard deviation (SD) as a scaling function [4,64]. After normalization and scaling, PCA itself was performed at a confidence level of 95%, thus generating both score and loading plots. The PCA of the NMR spectra resulted in a net variance of 82.34% distributed in the first two principal components (PCs; PC1 = 50.25% vs. PC2 = 32.09%).

**Figure 11:**
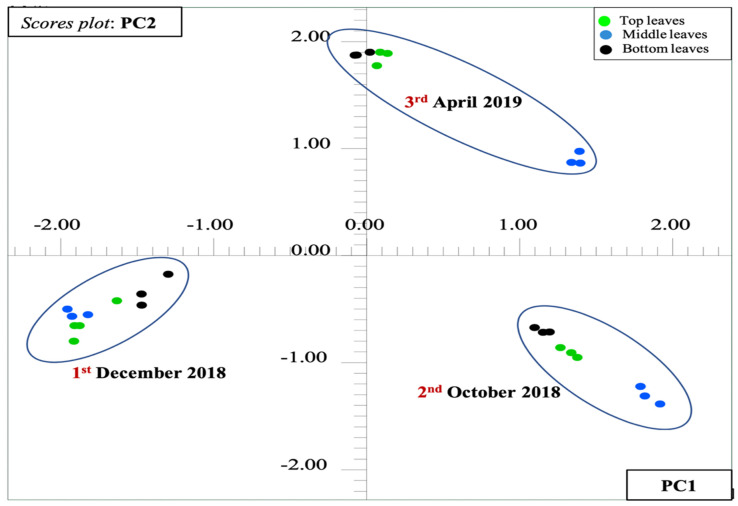
Scores plot from the principle component analysis (PCA) performed over ^1^H HR-MAS NMR spectra (ẟ 10.0–0.60, 0.04-ppm bucket size, and pareto-scaled) acquired directly from the leaves of *Berberis laurina* showing discrimination according to the season time. PC1 (50.25%) vs. PC2 (32.09%) of the 28 × 174 data matrix that revealed a net variance of 82.34%.

The inspection of the score plot permitted to visualize sample discriminations into three main groups or clusters over the time (October 2018, December 2018, and April 2019), although not between the leaf topology, instead (Figure 11). This means that the chemical variability in the course of time is higher than the variability due to the leaf topology. In other words, there is no highly significant differences in the chemical compositions of the top and bottom leaves. Rendering to the PCs, PC1 (50.25%) was responsible for the separation of the December 2018 and October 2018 samples, which were located along the negative and positive PC1, respectively, although both were along negative PC2. This found indicates that there a significant difference in the chemical composition of the leaves regarding the extreme periods (December to October), as previously observed by a visual inspection (see Section 2.3). On the other hand, the April 2019 samples were discriminated from the other groups only in PC2 (32.09%), being positive in PC2, although between positive and negative in PC1, which means they present a transition chemical composition.

**Figure 12:**
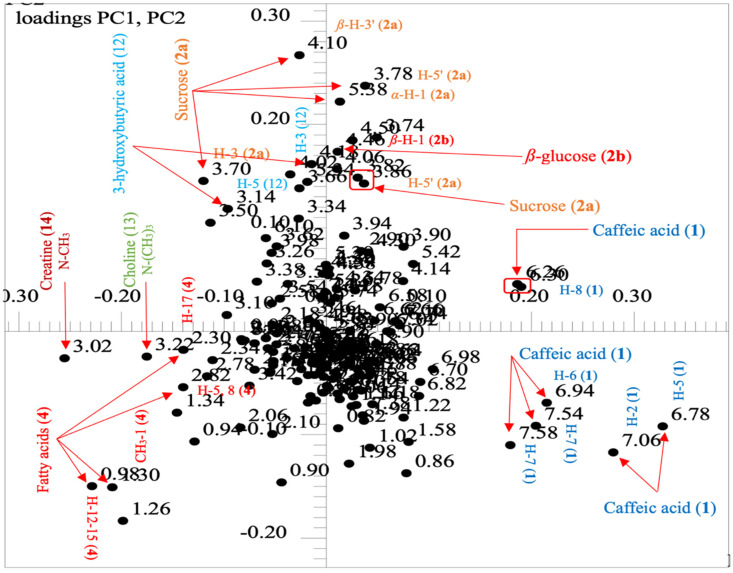
Loadings plot showing all chemical features for each group revealed in the scores plot, obtained with spectral widths of ẟ 10.0 to 0.60 utilizing the bucket size ẟ 0.04 and pareto-scaled PC1 (50.25%) vs. PC2 (32.09%) of 28 × 174 data matrix that revealed a net variance of 82.34%.

The main chemical features responsible for group separations in the course of time were achieved by looking in the same direction of the loadings plot (Figure 12) resulting from the PCA. Throughout, December 2018 samples were separated mainly based on three metabolites: fatty acids (**4**), choline (**13**), and creatine (**14**). In such, due to variables (i.e., NMR chemical shifts) at ẟ 0.98/0.96–1.00 (t7.6 Hz, H-1), a characteristic signal from a methyl group (CH_3_) of fatty acids in the ^1^H NMR spectra, as well as signals at ẟ 2.30/2.28-2.32 (m, H-17); 2.82/2.80-2.84 (m, H-5 and 8); and ẟ 1.30/1.28-1.32 (*brs*, H-12, 13, 14, and 15) of all methylene groups (CH_2_) from the fatty acids (**4**). The main discriminatory variables for the two other metabolites were two singlets, one from choline (**13**) at ẟ 3.22\3.20-3.24 (s) regarding the methyl hydrogen nuclei in N-(CH_3_)_3_ and the other from creatine (**14**) at ẟ 3.02\3.00-3.04 (s) for the hydrogen nuclei in a methyl group in N-CH_3_ in the molecular structure (Figure 12).

In the same way, October 2018 samples were discriminated almost exclusively due to a central metabolite, caffeic acid (**1**), supporting the visual section of the spectra (Section 2.3). In such, several signals of caffeic acid (**1**) appearing in different NMR chemical shifts, such as such those from a *trans*-configuration spin system at ẟ 7.58/7.56-7.60 (d 15.9 Hz, H-8), and its relative counterpart at ẟ 7.06/7.04-7.08 (d 1.9 Hz, H-2), as well as those at ẟ 6.78/6.76-6.80 (d 8.1 Hz, H-5) and ẟ 6.95/6.93-6.97 (dd 8.1 and 1.9 Hz, H-6) from two mutually *para*-coupled aromatic spin systems, were responsible for sample discriminations (Figure 12). Caffeic acid is functional metabolite including antipredator and leaf protector properties. Thus, its boost in biosynthesis may be associated by the need of plant defense purposes against microorganisms and other predators that increase with the humidity, since October 2018 was of high rainfall precipitation (Figure S12).

Finally, April 2019 samples were based on three molecular components: sucrose (**2a**), β-glucose (**2b**), and caffeic acid (**1**). Several NMR chemical shifts from **2a** at ẟ 5.38/5.36-5.40 (d 3.8 Hz, α-H-1), 4.10/4.08-4.12 (d 8.3 Hz, β-H-3’), and 3.78/3.76-3.80 (m, H-5’,), as well as the doublet at ẟ 4.47/4.45-4.49 (d 7.8 Hz, β-H-1) from **2b**, appeared to be the main ones responsible for sample discriminations in positive-PC2. On other hand, the remaining signals were from caffeic acid (**1**) in the range of ẟ 6.30/6.28-6.32 (d 15.9 Hz, H-8) from a hydrogen nuclei in *E*-configuration to H-7 in the molecular structure, correspondingly (Figure 12). By this, it can be concluded that the apices on caffeic acid (**1**) biosynthesis can be achieved during April to October, mainly in the later one.

Considering the weather conditions when samples were collected, it can be realized that there is a high correlation between group discriminations in the PCA and the season time. October 2018 was of high precipitation rates and low temperatures, while December 2018 presented high temperatures and a significant reduction in rainfall precipitation. April 2019 can be described as a transition period, with higher rainfall precipitation rates than December 2018 but lower than October. The same can be observed for the temperature; while December and October presented the highest and lowest temperatures, respectively, April was in between instead (Figure S12). This finding clearly supports that environmental conditions have significant influences on the chemical compositions in the leaves of *Berberis laurina*. Moreover, HR-MAS NMR proved to be a tool of choice in investigating plant tissues in their natural, unaltered states.

## 3. Experimental

### 3.1. Botanical Materials

Leaves (top, middle, and bottom); stem; and root samples (Figure 1) of *Berberis laurina* Billb. (Berberidaceae) species were collected during October 2018 to April 2019 from an open atmosphere in the Botanical Garden of Curitiba (Coordinates 25°26′27″ S, 49°14′24″ W: 910 m high), Curitiba, PR, Brazil. The plant was equally exposed to environmental interactions such as sunlight, moisture, airstream.

The taxonomical identification of the species was completed in the Herbarium of the Botanical Garden of Curitiba, PR, Brazil, and voucher specimen was deposited under the number MBM 415083 (Figure S14). All collected botanical material samples, including healthy leaves, stems, and roots, were first washed under running water to remove contamination. Followed by root and stem samples directly stored under freezing temperatures (−18 °C), while leaf samples were previously dried under circulating air for two days at an average temperature of 45 °C and then stored at −18 °C. The overall botanical material was then submitted to HR-MAS and liquid-state two-dimensional (2D) NMR analyses.

### 3.2. ^1^H HR-MAS NMR

To achieve high-resolution ^1^H HR-MAS NMR spectra, the leaves, roots, and stems were frozen in liquid nitrogen in a mortar and then grinded separately with aid of a pestle. After that, around 10 mg of the powder was inserted into a 50-µL zirconium oxide HR-MAS rotor followed by subjecting 40 µL of deuterated methanol (CD_3_OD, 99.8% D, TMS 0.05% (*v*/*v*) (Cambridge Isotopes Laboratory, Cambridge, MA, USA) for lock and shimming purposes. The botanical material in the HR-MAS rotor was mixed with solvent, and bubbles were removed with a syringe needle and homogenized; eventually, the rotor was tightly packed. Each individual sample was left in the solvent inside the HR-MAS rotor for about 15 min to swell and attain a gel-like state prior to HR-MAS NMR measurements.

^1^H HR-MAS NMR analyses were carried out on a Bruker AVANCE 400 NMR spectrometer (Bruker, Karlsruhe, Germany) operating at 9.4 Tesla (^1^H = 400.13 MHz). The spectrometer was equipped with a four channel (^1^H, ^13^C, ^15^N, and ^2^H (lock channel)) 4-mm HR-MAS probe with actively gradient field along the magic angle direction (Figure S15). The rotors were spun at 5 kHz under 296 K temperature. Similarly, radio frequency (RF) circuit was tuned-matched to the ^79^Br (≤ ^13^C = 100 MHz) frequency by using standard material (KBr) and realized that the magic angle was matching (θ = 54.74°). In the same way, the magnetic field (B_0_) was manually homogenized by adjusting *Z*, *Z^3^*, *X*, *XZ*, *XZ^2^*, and *XYZ* shim coils, and tuning-matching was performed to the hydrogen nuclei channel coil circuit to ^1^H frequency.

The ^1^H HR-MAS NMR experiments were performed with aid of the solvent suppression, *zgcppr* pulse sequence (Bruker library, Karlsruhe, Germany)), to manipulate the intense water resonance. Overall acquisition parameters used in *zgcppr* were included: free induction decay (FID) size (TD = 64 k data points), spectral width (SW = 8012.8 Hz), acquisition time (AQ = 4.09 s), FID resolution (FIDRES = 0.12 Hz), receiver gain (RG = 57), transmitter offset frequency (O1 = 1955.0 Hz), temperature (296 K), recycle delay (D1 = 1 s), presaturation power attenuation (pl9 = 55 dB), 90° flip angle pulse of 5.63 µs, and total utilized scans (NS = 256). All spectra were processed by applying an exponential window multiplication to the free induction decays (FIDs) using a Lorentzian line-broadening function (LB = 0.3) and zero-filled to 64 k data points.

### 3.3. Liquid-State (2D) NMR

Once the 1D NMR spectra acquired directly from in nature sample presented a high overlap of signals, the molecular structure identification in the samples were facilitated by performing 2D NMR experiments in a liquid state. For these, 300-mg powdered botanical material were weighed in a microcentrifuge tube (1000 µL) followed by an addition of 650-µL deuterated methanol (CD_3_OD), sonicated (25 °C, 40 min), centrifuged (30 min), and eventually, the supernatant was transferred into a 5-mm NMR tube.

The 2D NMR analyses were carried out on a Bruker AVANCE III 400 NMR spectrometer (Bruker, Karlsruhe, Germany) operating at 9.4 Tesla (^1^H = 400.13 MHz and ^13^C at 100.62 MHz). The spectrometer was equipped with a three-channel (^1^H, ^2^H (lock channel) and X-nucleus) 5-mm broad-band inverse detection probe with actively gradient field along z-direction.

The single bond (^1^*J*_H-C_ = 145 Hz) to long-range multiple bonds (^LR^*J*_H-C_ = 8 Hz) heteronuclear (^1^H-^13^C) correlation measurements were achieved through 2D multiplicity edited HSQC and HMBC NMR experiments. Additionally, the nearby and long-range homonuclear (^1^H-^1^H) correlation measurements were carried out by 2D COSY and TOCSY experiments. The importance of 2D multiplicity edited HSQC NMR was to simplify and differentiate CH and CH_3_ from CH_2_ groups in the molecules. The edited HSQC is an equivalent 2D pattern of the DEPT-135 experiment, which provides a multiplicities edition and correlation information to simplify intramolecular connections utilizing distinct phases (i.e., positive and negative phases). In this work, the blue (in the positive phase) represented CH and CH_3_, whereas the red color (in the negative phase) denoted all CH_2_ groups in the molecular structures.

### 3.4. Multivariate Statistical Analysis

Prior to multivariate statistical analysis, spectra base lines and phases were manually adjusted, and the NMR chemical shifts were referenced against the TMS signal at ẟ 0.00, as the internal reference, with the aid of Topspin software (Bruker). After that, the ^1^H HR-MAS NMR spectra (δ 0.60–10.00, except those regions regarding residual water signals (ẟ 4.73–5.00), as well as acetone (ẟ 2.14–2.16) and partially deuterated CD_3_OD-d_4_ (ẟ 3.29–3.32) signals) were binned into small segments of equal widths, providing 174 buckets (i.e., variables) 0.04-ppm wide, with the aid of AMIX software (Analysis of Mixtures software package, Bruker, Karlsruhe, Germany). The areas under each bucket were determined using the special integration mode from AMIX software and then normalized based on the total spectral area and pareto scaling. The buckets (i.e., NMR chemical shifts) were then used as input variables in the chemometric analysis by principal component analysis (PCA), a well-known unsupervised tool for multivariate data exploratory.

## 4. Conclusions

In this work, metabolite fingerprinting of *Berberis laurina* Billb. (Berberidaceae), a plant well-known for its diversity and pharmacological uses in traditional medicine since ancient times, was achieved for all three sections (leaves, roots, and stems) by means of HR-MAS NMR analysis. HR-MAS NMR-based fingerprinting allowed attaining chemical information directly from samples in their natural, unaltered states, preventing purification steps and preserving the expected status of all fingerprints in the samples and leading to highly reproducible comprehensive results. A total of 17 chemical compounds were identified, including caffeic acid, a recognized compound with plant protective properties, and berberine, a remarked alkaloid of the genus *Berberis* with manifold biological activities. Berberine was found in high amounts in roots, compared to stems and leaves, that, in turn, presented high amounts of caffeic acid (Figure S16). Additionally, a multivariate statistical analysis over HR-MAS NMR spectra from the leaves allowed to realize in a fast and simple way that there is an intrinsic correlation between the changes in the metabolic fingerprint and season time and environmental trend variabilities. All of these findings are supposed to be useful in understanding plant (bio)chemistry, metabolic events, medicinal purposes, health sciences, and genetic and biotechnological research fields.

## Figures and Tables

**Table 1:** High-Resolution Magic Angle Spinning Nuclear Magnetic Resonance (HR-MAS NMR) chemical shift assignments of all metabolites detected in leaves, roots, and stems of *Berberis laurina* Billb. (Berberidaceae).

Compound	Position	Current Work ^a^			Literature ^b^	
		δ_H_ (mult. *J*, Hz)	δ_C_	^LR^*J*_H-C_ (HMBC)	δ_H_ (mult. *J*, Hz)	δ_C_
Caffeic Acid (**1**)	**1**	-	127.8	-	-	127.2
	**2**	7.04 (d, 1.9)	115.2	149.5; 147.0; 122.7	7.07 (d, 2)	114.1
	**3**	-	147.0	-	-	145.5
	**4**	-	149.5	-	-	148.5
	**5**	6.76 (d, 8.1)	116.3	149.5; 147.0; 127.8; 122.7	6.77 (d, 7.8)	115.2
	**6**	6.95 (dd, 8.1;1.9)	122.7	149.5; 115.2	6.95 (dd, 7.9; 1.9)	121.7
	**7**	7.55 (d, 15.9)	146.8	168.8; 122.7; 115.2	7.62 (d, 16.1)	145.7
	**8**	6.27 (d, 15.9)	115.2	168.8; 127.8	6.42 (d, 16.1)	115.0
	**9**	-	168.8	-	-	167.8
Sucrose (**2a**)	**α-H-1**	5.38 (d, 3.8)	93.4	105.4; 74.5	5.37 (d, 3.8)	95.4
	**2**	3.42 (dd, 9.8; 3.8)	74.5	74.8	3.40 (dd, 9.8; 3.8)	75.0
	**3**	3.70 (t, 9.5)	74.8	71.3	3.68 (t, 9.6)	76.4
	**4**	3.36 (t, 9.5)	71.3	74.8; 71.6; 62.1	3.34 (t, 9.4)	73.0
	**5**	-	-	-	-	76.1
	**6**	-	-	-	3.70 (dd, 7.9; 4.0)	63.9
	**1’**	3.62 (d, 5.1)	63.8	105.4; 79.2	3.58 (d, 12.3)	65.7
	**2’**	-	105.4	-	-	107.1
	**β-H-3’**	4.10 (d, 8.3)	79.2	75.6; 63.8	4.08 (d, 8.2)	81.0
	**4’**	4.0 (m)	75.6	63.4	4.01 (t, 7.7)	77.4
	**5’**	3.69-3.87 (m)	83.9	83.9; 75.6	3.72-3.83	85.6
	**6’**	-	63.4	-	3.83-3.72	65.1
β-glucose (**2b**)	**β-H-1**	4.47 (d, 7.8)	98.2	-	4.45 (d, 7.8)	99.99
	**2**	3.11 (d, 7.8)	77.9	-	-	78.1
	**3**	-	74.6	-	-	79.8
	**4**	-	77.9	-	-	72.2
	**5**	-	74.6	-	-	79.9
	**6**	-	-	-	-	64.6
α-glucose (**2b**)	**α-H-1**	5.12 (d, 3.7)	93.5	-	5.09 (d, 3.7 Hz)	95.7
	**2**	3.36 (d, 3.7)	71.4	-	-	-
	**3**	-	-	-	-	-
	**4**	-	-	-	-	-
	**5**	-	-	-	-	-
	**6**	-	-	-	-	-
Threonine (**3**)	**1**	-	-	-	-	-
	**2**	-	-	-	3.51 (d, 12.0)	-
	**3**	4.29 (*br*, m)	-	-	4.27 (m)	-
	**4**	1.32 (d, 7.0)	30.2	-	1.32 (d, 7.0)	-
Fatty Acids (**4**)	**1**	0.97 (t, 7.6)	18.3	132.8	0.95 (t, 7.5)	-
	**2, 11**	2.1 (m)	28.1	129.2; 30.8	-	-
	**-HC = CH-**	5.34 (m)	129.3\72.0	26.6	-	-
	**5, 8**	2.81 (m)	26.3	129.2; 44.1	-	-
	**12-15**	1.30 (*br*, d)	30.5	30.5	-	-
	**16**	1.60 (m)	26.1	30.5	-	-
	**17**	2.32 (m)	35.2	174.8; 30.5; 26.1	-	-
	**18**	-	174.8	-	-	-
Arginine (**5**)	**1**	-	-	-	-	-
	**2**	3.27 (m)	71.4	-	3.25	-
	**3**	1.77 (m)	-	-	1.77	-
	**4**	1.60 (m)	26.0	-	1.59	-
	**5**	1.92 (m)	38.7	-	1.91	-
	**6**	-	-	-	-	-
Alanine (**6**)	**1**	-	-	-	-	-
	**2**	-	-	-	-	-
	**3**	1.48 (d, 7.20)	-	-	1.48 (d, 7.20)	-
3-hydorxybutyric acid (**7**)	**1**	-	-	-	-	-
	**2**	-	-	-	-	-
	**3**	4.18 (*brs*)	-	-	4.19	-
	**4**	1.21 (*brs*)	-	-	1.20	-
Valine (**8**)	**1**	-	-	-	-	-
	**2**	-	-	-	-	-
	**3**	-	-	-	2.27 (m)	-
	**4**	-	-	-	0.99 (d)	-
	**5**	1.03 (d, 2.7)	-	-	1.04 (d)	-
Trimethylamine (**9**)	**1**	2.90 (s)	40.2	-	2.89 (s)	-
Glutamic acid (**10**)	**1**	-	-	-	-	-
	**2**	2.45 (m)	-	-	2.37 (m)	-
	**3**	2.0 (m)	-	-	-	-
Fumaric acid (**11**)	**2,3**	6.54 (s)	120.9	-	6.52 (s)	-
Dihydroxy shikimate (**12**)	**1**	-	-	-	-	-
	**2**	6.38 (s)	115.4	127.8	6.39 (s)	-
	**3**	-	-	-	-	-
	**4**	-	127.8	-	-	-
	**5**	3.15-3.08 (m)	71.1	-	3.07 (m)	-
	**6**	2.66 (m)	63.5	192.5	2.62 (m)	-
	**7**	-	192.5	-	-	-
Choline (**13**)	**1**	3.22 (s) N-(CH_3_)_3_	55.0	77.8; 55.0	3.21 (s) N-(CH_3_)_3_	-
	**2**	-	77.8	-	-	-
	**3**	-	-	-	-	-
Creatine (**14**)	**-**	3.02 (s) N-CH_3_	43.9	-	-	-
Berberine (**15**)	**1**	7.63 (s)	107.7	152.1; 149.9; 139.6; 131.8	7.45 (s)	106.5
	**2**	-	152.1	-	-	152
	**2,3-OCH_2_O**	6.11 (s)	104.7	152.1; 149.9	6.13 (s, -OCH_3_)	103.6
	**3**	-	149.9	-	-	149.9
	**4a**	-	121.8	-	-	121.9
	**4**	6.96 (s)	110.7	152.1; 149.9; 121.8; 28.6	6.89 (s)	109.3
	**5**	3.26 (t, *J* = 6.3 Hz)	28.6	131.8; 121.8; 110.7; 58.3	3.26 (t, 5.6 Hz)	28.2
	**6**	4.92 (t, *J* = 6.3 Hz)	58.3	147.3; 139.6; 131.8; 28.6	4.95 (t, 5.6)	57.1
	**7**	-	-	-	-	-
	**8a**	-	135.3	-	-	135.1
	**8**	9.74 (s)	147.3	145.8; 139.6; 135.3; 58.3	9.78 (s)	146.4
	**9**	-	145.8	-	-	145.7
	**H_3_CO-9**	4.11 (s)	58.8	152.0	4.12 (s, -OCH_3_)	54.6
	**10**	-	152.0	-	-	152
	**H_3_CO-10**	4.20 (s)	63.6	145.8	4.35 (s, -OCH_3_)	62.5
	**11**	8.11 (d, 9.1 Hz)	129.3	145.8; 135.3	8.00 (d, 7.98 Hz)	128
	**12a**	-	123.3	-	-	123.3
	**12**	8.0 (d, 9.1 Hz)	125.4	152.0; 123.3	7.95 (d, 7.98 Hz)	124.5
	**13**	8.65 (s)	122.7	139.6; 125.4; 123.3; 122.7	8.61 (s)	121.5
	**14a**	-	131.8	-	-	131.9
	**14**	-	139.6	-	-	139.6

δ_H_ = ^1^H Nuclear Magnetic Resonance chemical shift, δ_C_ = ^13^C Nuclear Magnetic Resonance chemical shift, (mult. *J*, Hz) = Multiplicity and coupling constants in Hertz, ^LR^*J*_H-C_ (HMBC) = Long-range ^1^H-^13^C correlation from Heteronuclear Multiple-Bond Correlation. ^a^ Experimental work, acquired at 400 and 100-MHz for ^1^H and ^13^C, respectively, from swollen materials in CD_3_OD containing TMS (*v*/*v*, 0.05%) as the internal reference. (**1**) **^b^** ^1^H 500.13 and ^13^C 125.75 MHz in CD_3_OD [36]; (**2a-c**) ^b 1^H 800 and 201 MHz for ^1^H and ^13^C in CD_3_OD [46]; (**3**) ^b^, (**5**) ^b^, (**7**) ^b^, and (**8**) ^b 1^H 500.13-MHz in CD_3_OD, KH_2_PO_4_ in D_2_O and TSP [47]; (**4**) ^b 1^H 500.13-MHz in CD_3_OD, KH_2_PO_4_ in D_2_O and TSP [51]; (**6**) ^b 1^H 500.13 MHz in CD_3_OD + phosphate buffer in D_2_O [52]; (**9**) ^b^, (**10**) ^b^, (**11**) ^b^, (**12**) ^b^, and (**13**) ^b 1^H 800-MHz in D_2_O and TSP [40]; and (**14**) ^b^* and (**15**) ^b 1^H 400: ^13^C 100-MHz CD_3_OD [44]. The respective multiplicities are shown with the letters “s” (singlet), “d” (doublet), “t” (triplet), and “m” (multiplet). b* = confirmed with online databases.

## Supplementary Materials

The following are available online, Figure S1: Comparative 1H NMR spectra (400 MHz) of plant extract (CD3OD solution) and its respective natural state (~10 mg swollen in 40 µL CD3OD) from the leaves of Berberis laurina, Figure S2: Representative 1H HR-MAS NMR spectra acquired directly from different parts of Berberis laurina (400 MHz, ~10 mg swollen in 40 µL CD3OD), Figure S3: 1H-13C direct correlation map from multiplicity edited HSQC NMR experiment (ẟ 6.00–7.65 vs. ẟ 110.0–150.0) acquired from leaves of Berberis laurina (400 MHz, CD3OD). The labels refer to the assignments of intense correlation for compounds as indicate in brackets, Figure S4: 1H-13C direct correlation map from multiplicity edited HSQC NMR experiment (ẟ 3.00–5.45 vs. ẟ 30.0–140.0) recorded from leaves of Berberis laurina (400 MHz, CD3OD). The labels refer to the assignments of intense correlation for compounds as indicate in brackets, Figure S5: 1H-13C direct correlation map from multiplicity edited HSQC NMR experiment (ẟ 0.60–3.00 vs. ẟ 10.0–50.0) recorded from leaves of Berberis laurina (400 MHz, CD3OD). The labels refer to the assignments of intense correlation for compounds as indicate in brackets, Figure S6: 1H-13C long-range correlation map from HMBC NMR experiment (ẟ 0.50–8.00 vs. ẟ 5.0–190.0) recorded from leaves of Berberis laurina (400 MHz, CD3OD). The labels refer to the assignments of intense correlation for compounds as indicate in brackets, Figure S7: 1H-1H correlation map from COSY NMR experiment (ẟ 0.50–8.00 vs. ẟ 0.50–8.00) recorded from leaves of Berberis laurina (400 MHz, CD3OD), Figure S8: 1H-1H correlation map from TOCSY NMR experiment (ẟ 0.50–8.00 vs. ẟ 0.50–8.00) recorded from leaves of Berberis laurina (400 MHz, CD3OD), Figure S9: 1H-13C direct correlation map from multiplicity edited HSQC NMR experiment (ẟ 2.96–10.0 vs. ẟ 25.0–155.0) recorded from roots of Berberis laurina (400 MHz, CD3OD). The labels refer to the assignments of intense correlation for compounds as indicate in brackets, Figure S10. 1H-13C long-range correlation map from HMBC NMR experiment (ẟ -1.00–12.00 vs. ẟ -0.5–190.0) recorded from roots of Berberis laurina (400 MHz, CD3OD), Figure S11: 1H-1H correlation map from COSY NMR experiment (ẟ -0.50–10.10 vs. ẟ -0.50–10.10) recorded from roots of Berberis laurina (400 MHz, CD3OD), Figure S12: Climate data from September 2018 to April 2019 in Curitiba, PR, Brazil. Data source: INMET available at http://www.inmet.gov.br/, Figure S13: Boxplot regarding signal-to-noise (Y-scale) showing content variability for some compounds over time in the leaves of Berberis laurina, Figure S14: Botanical information associated to the species Berberis laurina Billb. (Berberidaceae), Figure S15: Schematic representation of applied technology in the current work. This includes a 4-mm HR-MAS rotor containing the sample and its transfer into a NMR spectrometer equipped with and 4-mm HR-MAS probe, in which sample is analyzed under the magic angle direction (54.74o) at moderate spinning speed (5 kHz), Figure S16: A pictorial representation regarding signal-to-noise (S/N) relationships associated to the berberine (15) and caffeic acid (1) contents in leaves (top-bottom), stem, and roots of Berberis laurina.
