# Supplementary material for: High-Resolution Magic Angle Spinning (HR-MAS) NMR-Based Fingerprints Determination in the Medicinal Plant Berberis laurina
Source: Molecules. 2020 Aug 11;25(16):3647. doi: 10.3390/molecules25163647 (PMC7465263; doi:10.3390/molecules25163647)
Supplement: Supplementary file 1 [file molecules-25-03647-s001.docx]

High-Resolution Magic Angle Spinning (HR-MAS) NMR-Based Fingerprints Determination in the Medicinal Plant *Berberis laurina*

Sher Ali ^1,^*, Gul Badshah ^1^, Caroline Da Ros Montes D’Oca ^1^, Francinete Ramos Campos ^2^, Noemi Nagata ^1^, Ajmir Khan ^3,4^, Maria de Fátima Costa Santos ^1^ and Andersson Barison ^1,^*

^1^ NMR Lab, Department of Chemistry, Federal University of Paraná, Curitiba, 81530-900, PR, Brazil

^2^ Department of Pharmacy, Federal University of Paraná, 80210-170, PR, Brazil

^3^ School of Packaging, Michigan State University, East Lansing, 48824-1223, MI, United States of America.

^4^ Institute of Chemistry, University of São Paulo, São Paulo, 05508-000, SP, Brazil.

***** Correspondence: Sher Ali alisherufpr@gmail.com and Andersson Barison, anderbarison@gmail.com

^1^H NMR (liquid-state)


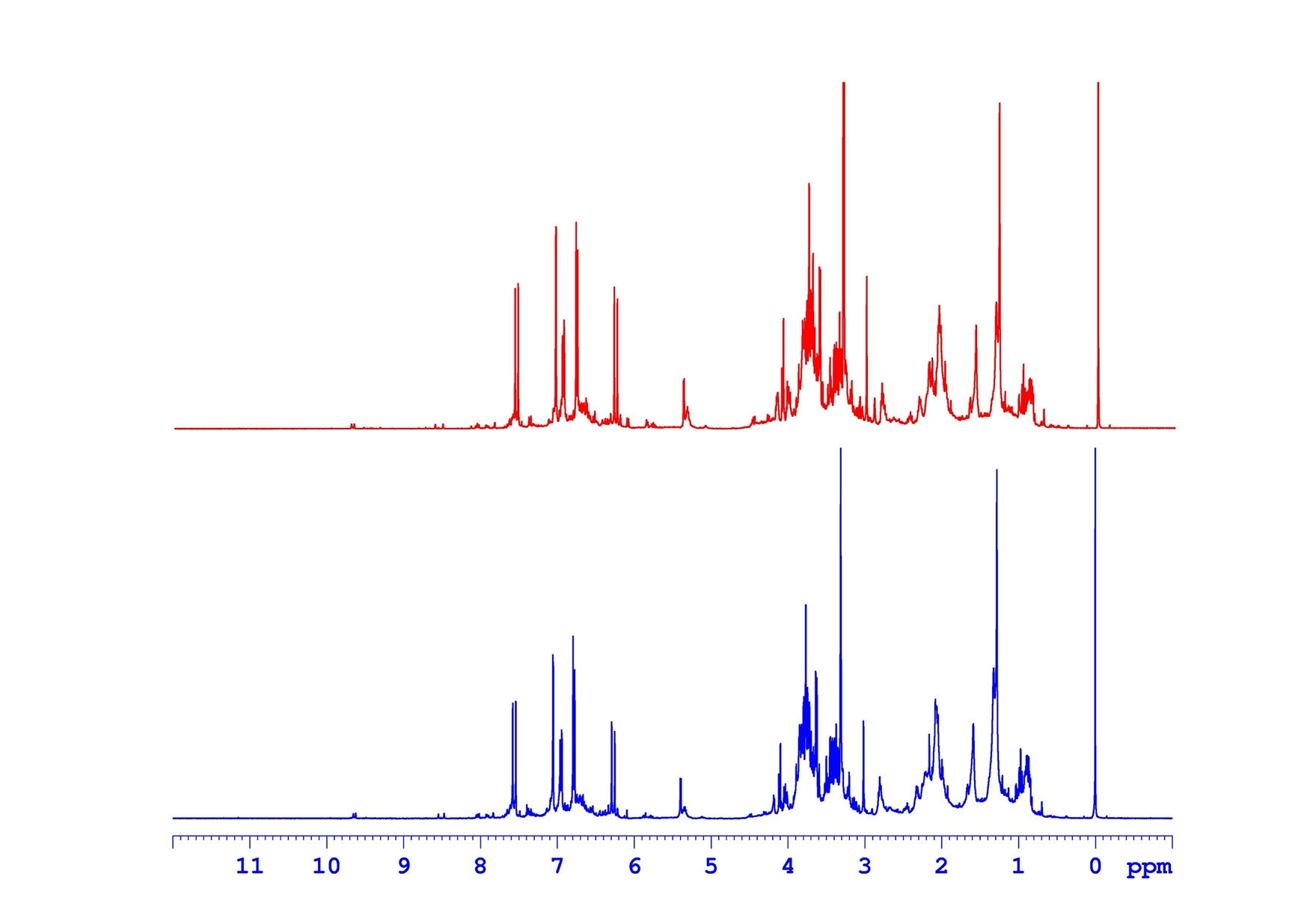


^1^H HR-MAS NMR (semisolid-state)

**Figure S1.** Comparative ^1^H NMR spectra (400 MHz) of plant extract (CD_3_OD solution) and its respective natural state (~10 mg swollen in 40 µL CD_3_OD) from the leaves of Berberis laurina.

Leaves

Stems

Roots

**Figure S2.** Representative ^1^H HR-MAS NMR spectra acquired directly from different parts of Berberis laurina (400 MHz, ~10 mg swollen in 40 µL CD_3_OD).

**Overview of homo- and heteronuclear correlation maps from 2D NMR experiments, which were performed in liquid-state from the crude extracts. 300 mg powder were extract with 650 µL of CD_3_OD.**


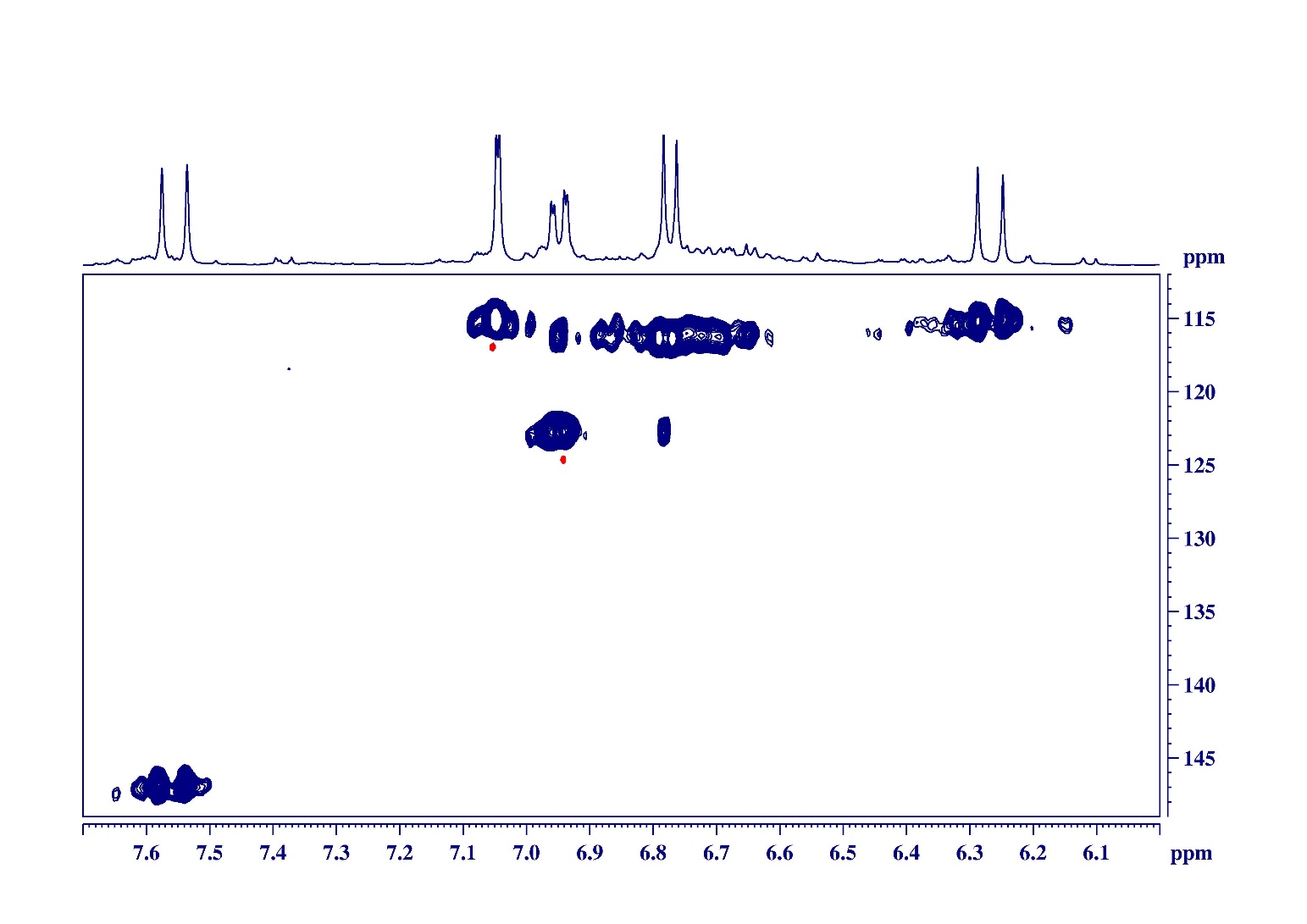


CH-7 (**1**):

ẟ_C_ =146.8; ẟ_H_ = 7.55

CH-8 (**1**):

ẟ_C_ =115.2; ẟ_H_ = 6.27

CH-2 (**12**):

ẟ_C_ =115.4; ẟ_H_ = 6.38

CH-5 (**1**):

ẟ_C_ =116.3; ẟ_H_ = 6.76

CH-6 (**1**):

ẟ_C_ =122.7; ẟ_H_ = 6.95

CH-2 (**1**):

ẟ_C_ =115.2; ẟ_H_ = 7.04

**Figure S3.** ^1^H-^13^C direct correlation map from multiplicity edited HSQC NMR experiment (ẟ 6.00–7.65 vs ẟ 110.0–150.0) acquired from leaves of Berberis laurina (400 MHz, CD_3_OD). The labels refer to the assignments of intense correlation for compounds as indicate in brackets.


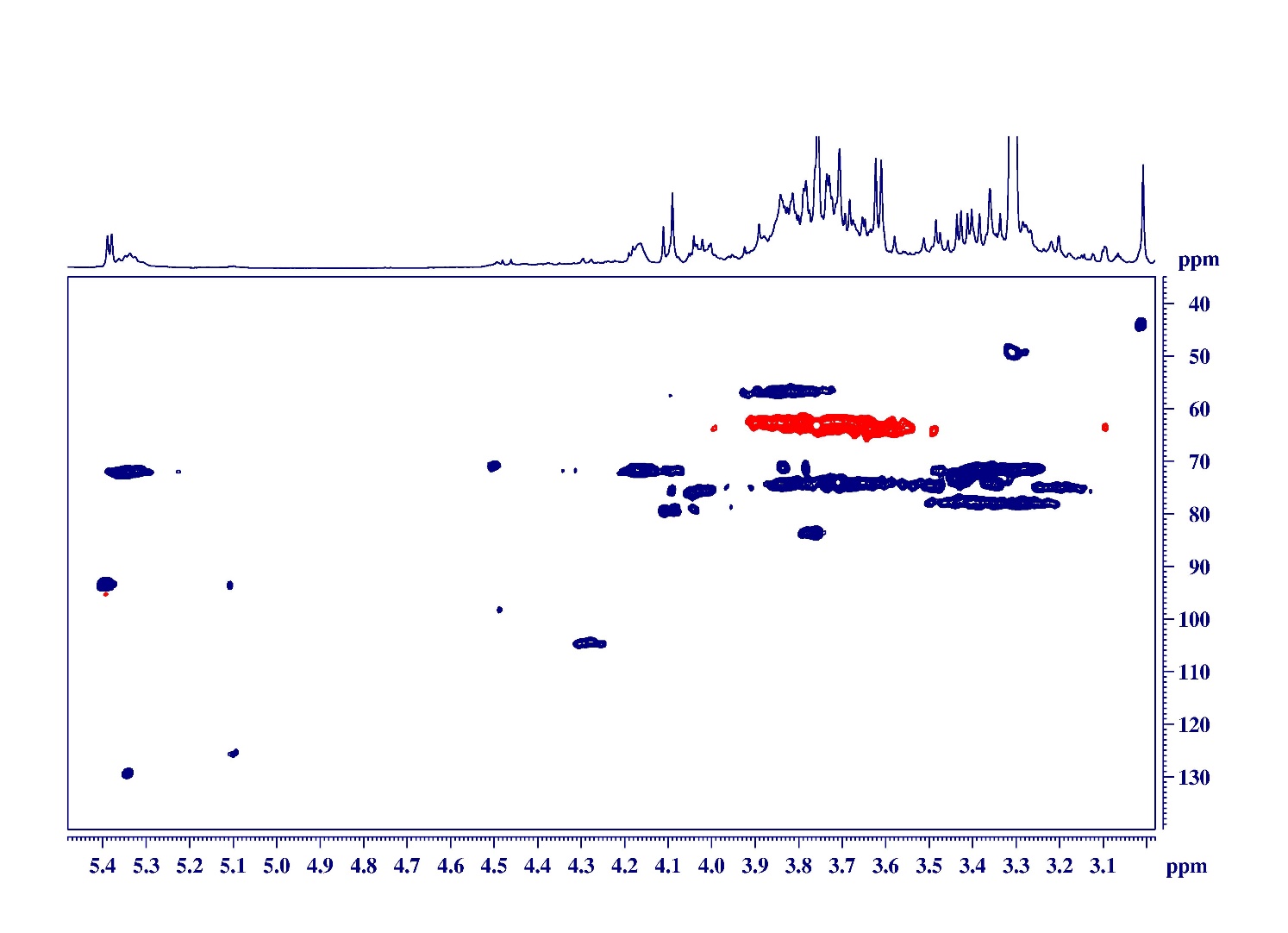


-HC=CH- (**4**):

ẟ_C_ =129.3; ẟ_H_ = 5.34

*α*-CH (**2a**):

ẟ_C_ =93.4; ẟ_H_ = 5.38

CH-3 (**7**):

ẟ_C_ = x; ẟ_H_ = 4.18

N-CH_3_ (**14**):

ẟ_C_ = 43.9; ẟ_H_ = 3.0

CH-4 (**2b**):

ẟ_C_ = 77.9; ẟ_H_ = x

CH-2, 4 (**2a**); CH-3, 5 (**2b**):

ẟ_C_ = 74.5-71.3;

ẟ_H_ = 3.42-3.36

CH-2(**5**):

ẟ_C_ = 71.4; ẟ_H_ = 3.27

CH-2 (**2b**):

ẟ_C_ = 77.9; ẟ_H_ = 3.11

CH-5′, CH-3 (**2a**):

ẟ_C_ = 83.39; ẟ_H_ = 3.87-3.69

-HC=CH- (**4**):

ẟ_C_ =72.0; ẟ_H_ = 5.34

*α*-CH (**2c**):

ẟ_C_ =93.5; ẟ_H_ = 5.12

CH-4′ (**2a**):

ẟ_C_ = 75.6; ẟ_H_ = 4.0

*β*-CH (**2b**):

ẟ_C_ =98.2; ẟ_H_ = 4.47

**Figure S4.** ^1^H-^13^C direct correlation map from multiplicity edited HSQC NMR experiment (ẟ 3.00–5.45 vs ẟ 30.0–140.0) recorded from leaves of Berberis laurina (400 MHz, CD_3_OD). The labels refer to the assignments of intense correlation for compounds as indicate in brackets.


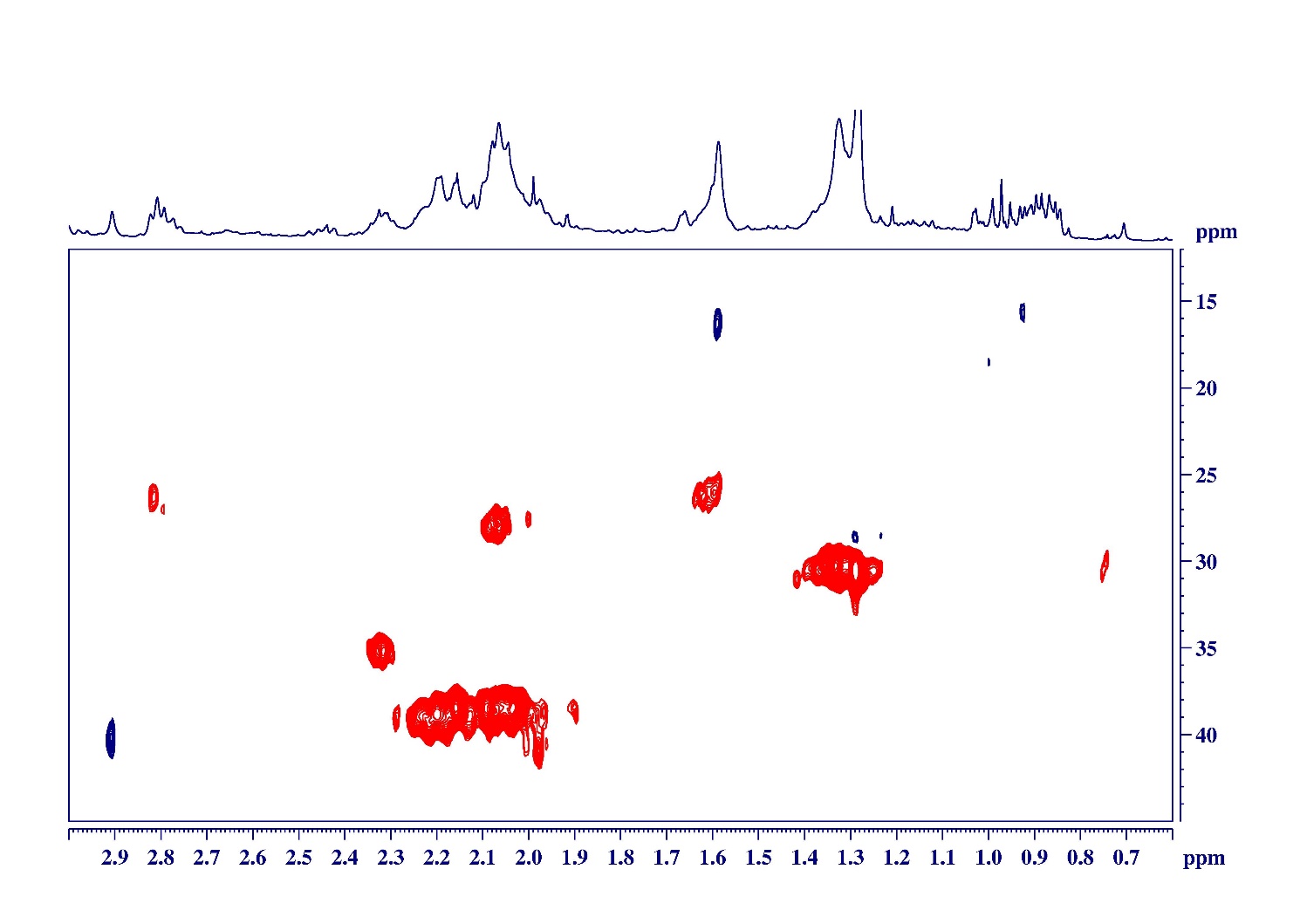


CH_2_-5 (**5**): ẟ_C_ = 38.7; ẟ_H_ = 1.92

CH_2_-3 (**10**): ẟ_C_ = x; ẟ_H_ = 2.00

CH_2_-4 (**5**):

ẟ_C_ = 26.0; ẟ_H_ = 1.60

CH_2_-16 (**4**):

ẟ_C_ = 26.1; ẟ_H_ = 1.60

CH_2_-5, 8 (**4**): ẟ_C_ = 26.3; ẟ_H_ = 2.81

CH_3_ (**9**): ẟ_C_ = 40.2; ẟ_H_ = 2.90

CH_2_-17 (**4**): ẟ_C_ = 35.2; ẟ_H_ = 2.32

CH_2_-2, 11 (**4**): ẟ_C_ = 28.1; ẟ_H_ = 2.1

**Figure S5.** ^1^H-^13^C direct correlation map from multiplicity edited HSQC NMR experiment (ẟ 0.60–3.00 vs ẟ 10.0–50.0) recorded from leaves of Berberis laurina (400 MHz, CD_3_OD). The labels refer to the assignments of intense correlation for compounds as indicate in brackets.

C-9: ẟ_C_ = 168.8 (**1**)

C-6: ẟ_C_ = 122.7 (**1**)

C-2: ẟ_C_ = 115.2 (**1**)

C-4: ẟ_C_ = 127.8 (**12**)

C-2: ẟ_C_ = 115.2 (**1**)

C-6: ẟ_C_ = 122.7 (**1**)

C-3: ẟ_C_ = 74.8 (**2a**)

C-2': ẟ_C_ = 105.4 (**2a**)

C-4': ẟ_C_ = 75.6 (**2a**)

C-1: ẟ_C_ = 127.8 (**1**)

C-6: ẟ_C_ = 122.7 (**1**);

C-1: ẟ_C_ = 127.8 (**1**).

C-3: ẟ_C_ = 147.0 (**1**);

C-4: ẟ_C_ = 149.5 (**1**).

C-18: ẟ_C_ = 75.8 (**4**)

C-3,4,6,7,9,10: ẟ_C_ = 129.3 (**4**)

C-12-15: ẟ_C_ = 30.5 (**4**)

C-16: ẟ_C_ = 26.3 (**4**);

C-12-15: ẟ_C_ = 30.5 (**4**).

C-5,8: ẟ_C_ = 26.3 (**4**)

**Figure S6.** ^1^H-^13^C long-range correlation map from HMBC NMR experiment (ẟ 0.50–8.00 vs ẟ 5.0–190.0) recorded from leaves of Berberis laurina (400 MHz, CD_3_OD). The labels refer to the assignments of intense correlation for compounds as indicate in brackets.

H-7 (**1**)

H-8 (**1**)

H-5 (**1**)

H-6 (**1**)

β-H-1 (**2a**)

H-2 (**2a**)

H-5,8 (**4**)

H-3,4,6,7,9,10 (**4**)

α-H-1 (**2c**)

H-2 (**2c**)

β-H-1 (**2b**)

H-2 (**2b**)

H-3,4,6,7,9,10 (**4**)

H-2,11 (**4**)

H-17 (**4**)

H-16 (**4**)

H-2,11 (**4**)

H-12-15 (**4**)

H-1 (**4**)

**Figure S7.** ^1^H-^1^H correlation map from COSY NMR experiment (ẟ 0.50–8.00 vs ẟ 0.50–8.00) recorded from leaves of Berberis laurina (400 MHz, CD_3_OD).

**Figure S8.** ^1^H-^1^H correlation map from TOCSY NMR experiment (ẟ 0.50–8.00 vs ẟ 0.50–8.00) recorded from leaves of Berberis laurina (400 MHz, CD_3_OD).

CH-8 (**15**): ẟ_C_ =147.3; ẟ_H_ = 9.74

CH-6' (**2a**):

ẟ_C_ =63.4; ẟ_H_ = x

CH-11 (**15**):

ẟ_C_ =129.3; ẟ_H_ = 8.11

CH-1 (**2a**):

ẟ_C_ =94.6; ẟ_H_ = 5.38

-OCH_3_-10 (**15**):

ẟ_C_ =63.3; ẟ_H_ = 4.20

-OCH_3_-9 (**15**):

ẟ_C_ =58.8; ẟ_H_ = 4.11

CH-4' (**2a**):

ẟ_C_=76.9; ẟ_H_ = 4.0

CH-3' (**2a**):

ẟ_C_=80.4; ẟ_H_ = 4.10

CH-5' (**2a**): ẟ_C_=84.9;

ẟ_H_ = 3.87-3.69

CH-12 (**15**):

ẟ_C_ =125.4; ẟ_H_ = 8.0

CH-1 (**15**):

ẟ_C_ =107.7; ẟ_H_ = 7.63

CH-6 (**15**):

ẟ_C_ =58.3; ẟ_H_ = 4.92

2,3-OCH_2_O-12 (**15**):

ẟ_C_ =104.7; ẟ_H_ = 6.11

CH-13 (**15**):

ẟ_C_ =122.3; ẟ_H_ = 8.65

CH-4 (**15**):

ẟ_C_ =110.7; ẟ_H_ = 6.66

**Figure S9.** ^1^H-^13^C direct correlation map from multiplicity edited HSQC NMR experiment (ẟ 2.96*–*10.0 *vs* ẟ 25.0*–*155.0) recorded from roots of *Berberis laurina* (400 MHz, CD_3_OD). The labels refer to the assignments of intense correlation for compounds as indicate in brackets.

C-13: ẟ_C_ = 122.7 (**15**);

C-12a: ẟ_C_ = 123.3 (**15**);

C-12: ẟ_C_ = 125.4 (**15**).

C-14a: ẟ_C_ = 131.8 (**15**)

C-12a: ẟ_C_ = 123.3 (**15**)

C-8a: ẟ_C_ = 135.3 (**15**)

C-14: ẟ_C_ =139.6 (**15**)

C-14: ẟ_C_ = 139.6 (**15**)

C-9: ẟ_C_ = 145.8 (**15**)

C-9: ẟ_C_ = 145.8 (**15**)

C-10: ẟ_C_ = 152.0 (**15**)

C-3: ẟ_C_ = 149.9 (**15**);

C-2: ẟ_C_ = 152.1 (**15**).

C-4: ẟ_C_ = 110.7 (**15**)

C-4a: ẟ_C_ = 121.8 (**15**)

C-14a: ẟ_C_ = 131.8 (**15**)

C-9: ẟ_C_ = 145.8 (**15**)

C-10: ẟ_C_ = 152.0 (**15**)

C-8: ẟ_C_ = 147.3 (**15**)

C-4a: ẟ_C_ = 121.8 (**15**)

C-14: ẟ_C_ = 139.6 (**15**)

C-6: ẟ_C_ = 58.3 (**15**)

C-6: ẟ_C_ = 58.3 (**15**)

C-5: ẟ_C_ = 28.6 (**15**)

C-6: ẟ_C_ = 58.3 (**15**)

**Figure S10.** ^1^H-^13^C long-range correlation map from HMBC NMR experiment (ẟ -1.00–12.00 vs ẟ -0.5–190.0) recorded from roots of Berberis laurina (400 MHz, CD_3_OD).

H-6 (**15**)

H-11 (**15**)

H-12 (**15**)

H-5 (**15**)

**Figure S11.** ^1^H-^1^H correlation map from COSY NMR experiment (ẟ -0.50*–*10.10 *vs* ẟ -0.50*–*10.10) recorded from roots of *Berberis laurina* (400 MHz, CD_3_OD).

**Figure S12.** Climate data from September 2018 to April 2019 in Curitiba, PR, Brazil. Data source: INMET available at http://www.inmet.gov.br/.

Leaves topology

Middle

Top

Bottom

**Figure S13**. Boxplot regarding signal-to-noise (Y-scale) showing content variability for some compounds over time in the leaves of *Berberis laurina*.


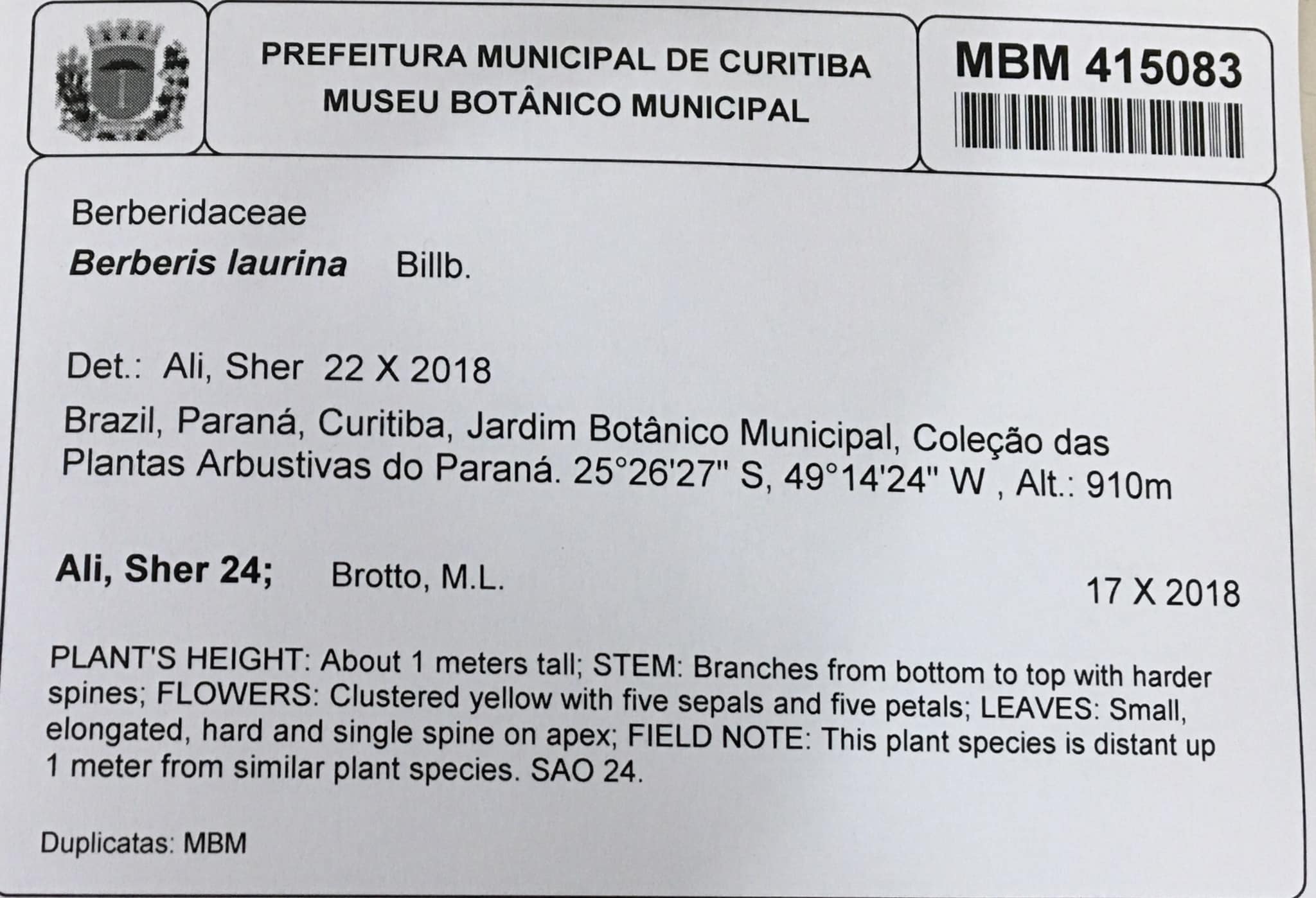


**Figure S14**. Botanical information associated to the species Berberis laurina Billb. (Berberidaceae).


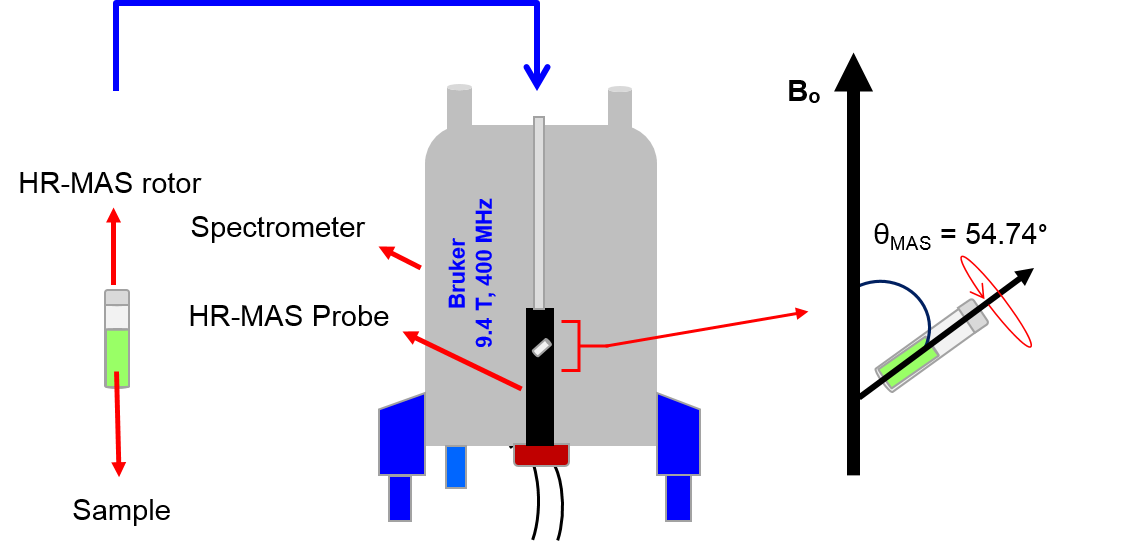


**Figure S15.** Schematic representation of applied technology in the current work. This includes a 4-mm HR-MAS rotor containing the sample and its transfer into a NMR spectrometer equipped with and 4-mm HR-MAS probe, in which sample is analyzed under the magic angle direction (54.74^o^) at moderate spinning speed (5 kHz).

^1^H NMR (liquid-state)

^1^H HR-MAS NMR (semisolid-state)


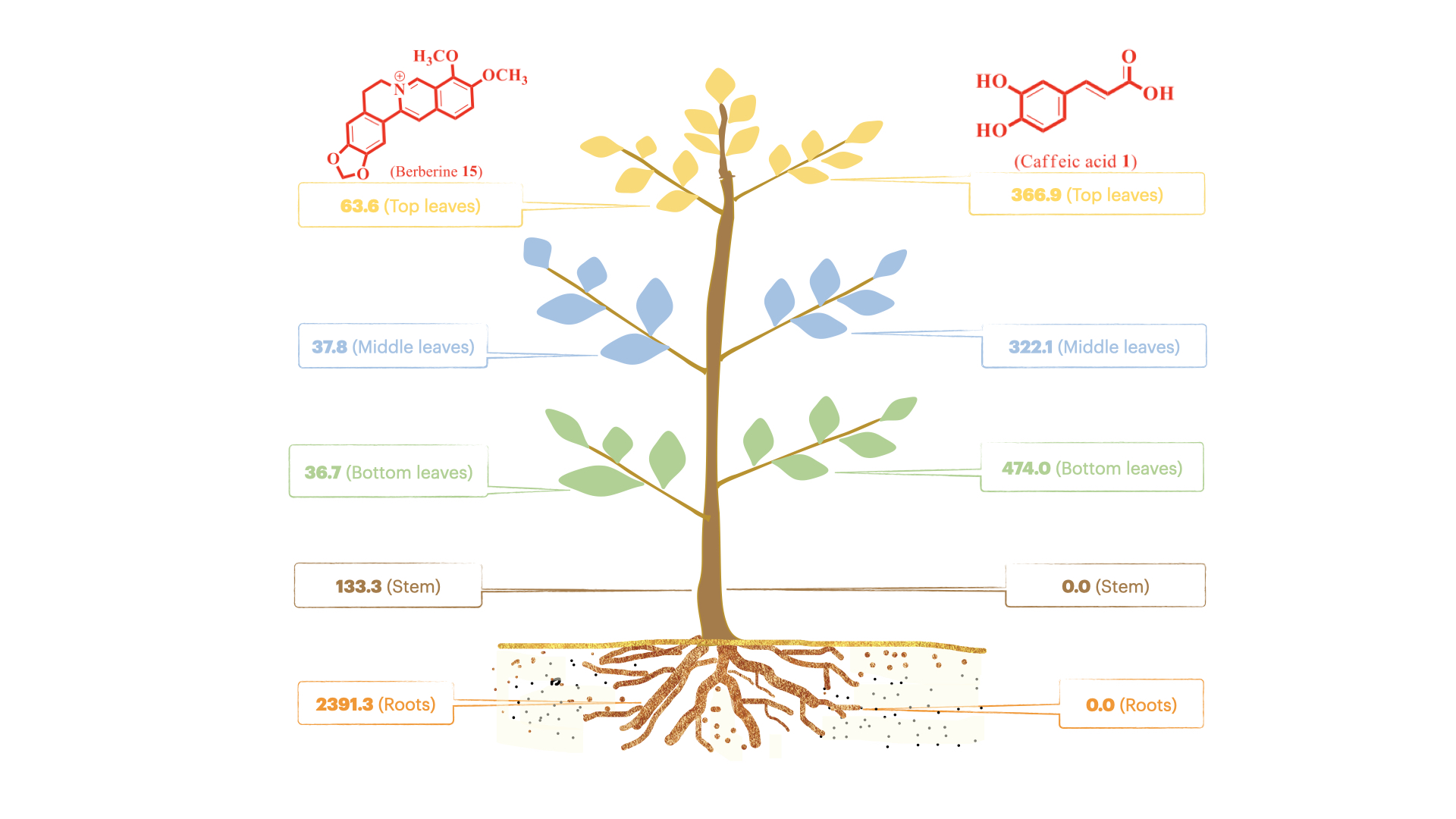


**Figure S16**. A pictorial representation regarding signal-to-noise (*S/N*) relationships associated to the berberine (**15**) and caffeic acid (**1**) contents in leaves (top-bottom), stem, and roots of *Berberis laurina*.
